# Timekeepers of the gut: host circadian rhythms and microbial modulators

**DOI:** 10.1093/lifemeta/loag003

**Published:** 2026-01-30

**Authors:** Xianda Ma, Qiyi Yu, Zheng Kuang

**Affiliations:** Department of Biological Sciences, Carnegie Mellon University, Pittsburgh, PA 15213, United States; Department of Biological Sciences, Carnegie Mellon University, Pittsburgh, PA 15213, United States; Department of Biological Sciences, Carnegie Mellon University, Pittsburgh, PA 15213, United States

**Keywords:** circadian rhythms, intestine, gut microbiota, digestive diseases, microbial metabolites

## Abstract

The intestine is more than a digestive organ. It is a system under circadian control, where cellular renewal, barrier integrity, absorption, immunity, and microbial ecology are orchestrated in time. Emerging evidence reveals that circadian rhythms not only regulate the daily turnover of intestinal epithelium but also fine-tune digestive enzyme expression, mucosal defense, and gut hormone secretion. These processes are driven by core clock proteins and synchronized by feeding cues, neural signals, and microbial metabolites. The gut microbiota consists of essential symbionts that themselves exhibit diurnal oscillations in composition and function, in turn feeding back to modulate host circadian pathways. Disruption of this host–microbiota temporal alignment, as occurs with jet lag, shift work, or high-fat diets, impairs intestinal homeostasis and elevates risk for inflammation, infection, and metabolic disorders. This review integrates evidence from mouse, zebrafish, fly, and human studies to highlight the rhythmic regulation of gut physiology, emphasizing how coordination between the host clock and microbiota sustains health. Viewing the gut as a circadian conductor underscores new opportunities for chronotherapy and microbiota-targeted interventions.

## Circadian rhythms

Circadian rhythms are intrinsic time-regulating mechanisms that enable organisms to adapt to the 24-h rotational cycle of Earth, driving physiological and behavioral state changes from unicellular organisms to mammals [1]. Diurnal oscillations are coordinated by central pacemakers, such as the suprachiasmatic nucleus (SCN) of the hypothalamus in mammals or clusters of clock neurons in the brain of *Drosophila*. These central clocks work in concert with peripheral clocks distributed across organs and tissues, together entrained by external zeitgebers (environmental time cues that synchronize circadian rhythms) [2]. Core clock proteins, including CLOCK, basic helix-loop-helix ARNT-like protein 1 (BMAL1), period (PER), and cryptochrome (CRY), regulate rhythmic oscillations within the SCN through the transcription-translation feedback loop (TTFL) [1]. Moreover, these clock components are widely expressed in peripheral organs such as the liver, muscle, adipose tissue, and intestine, orchestrating rhythmic oscillations in metabolism, immune function, and hormone secretion [3].

Environmental zeitgebers, including light, temperature, diet, sleep, physical activity, and social behaviors, play a crucial role in regulating circadian rhythms [4]. In circadian research, light–dark cycles are commonly expressed using zeitgeber time (ZT), with ZT0 defined as lights-on and ZT12 as lights-off. In addition to these relatively stable external cues, the gut microbiota and its metabolites have emerged as dynamic modulators of circadian rhythms. It not only exhibits endogenous oscillations but also responds to behavioral cues from the host [5]. The gut, as a major peri­pheral circadian oscillator, integrates signals from environmental zeitgebers to regulate the rhythmicity of its core physiological functions, including epithelial cell turnover, nutrient digestion and absorption, intestinal motility, hormone secretion, and immune responses. Beyond its local functions, the gut also plays a role in synchronizing circadian rhythms in distal organs such as the brain, liver, adipose tissue, heart, and pancreas. Disruption of gut circadian rhythms can impair physiological homeostasis, heighten vulnerability to infection and inflammation, and drive metabolic and neurological pathologies [6–8]. Thus, understanding how circadian rhythms regulate gut physiology and how gut microbes mediate host–environment crosstalk is central to developing novel therapeutic and preventive interventions.

**Figure 1:**
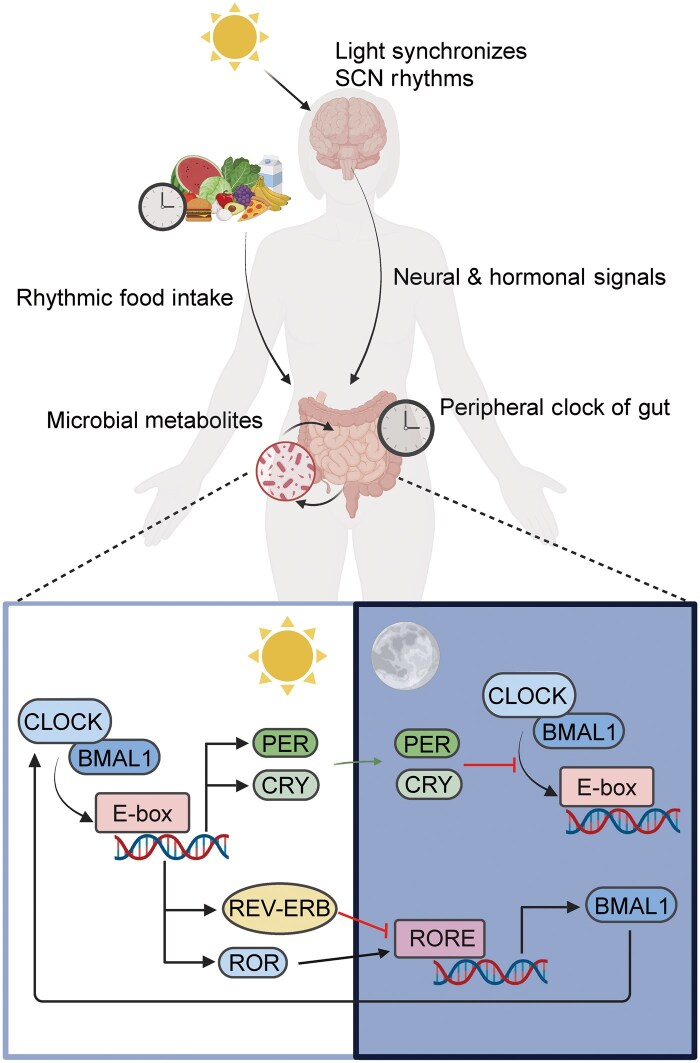
Circadian regulation in the gut. The top panel illustrates how environmental zeitgebers synchronize gut circadian rhythms. Light entrains the central clock located in the SCN, which regulates peripheral clocks via neural signals, cortisol, and melatonin. Feeding rhythms and microbial metabolites also influence the intestinal clock. The bottom panel depicts the core molecular clock mechanism within gut cells: during the day, CLOCK and BMAL1 form a heterodimer that binds to E-box elements to drive the transcription of *Per*, *Cry*, *Rev-erb*, and *Ror*. At night, the PER/CRY complex inhibits CLOCK:BMAL1 activity, while REV-ERB represses and ROR activates BMAL1 expression via RORE elements. This TTFL sustains circadian oscillations that coordinate rhythmic functions such as epithelial renewal, barrier integrity, nutrient absorption, and immune responses in the gut.

A central part of circadian regulation is a conserved molecular clock system that controls rhythmic gene expression in both the brain and other body tissues. At the molecular level, the core clock proteins BMAL1 and CLOCK form a heterodimer that binds to E-box elements in the promoter regions of target genes, driving the transcription of clock-controlled genes, including *Per*, *Cry*, reverse erythroblastosis virus (*Rev-Erb*), and retinoic acid receptor-related orphan receptor (*Ror*) [1] (Fig. 1). PER and CRY proteins accumulate and form complexes that translocate into the nucleus to inhibit BMAL1-CLOCK activity, establishing a nega­tive feedback loop in the circadian clock. Meanwhile, REV-ERB and ROR regulate the transcription of *Bmal1* via ROR response elements (ROREs), forming an auxiliary loop that stabilizes the oscillation. This tightly regulated feedback architecture allows the circadian clock to drive rhythmic expression of downstream genes. These molecular mechanisms provide the foundation for rhythmic regulation of physiological functions throughout the body, including the gut.

## Functions of circadian rhythms in the gut

**Figure 2:**
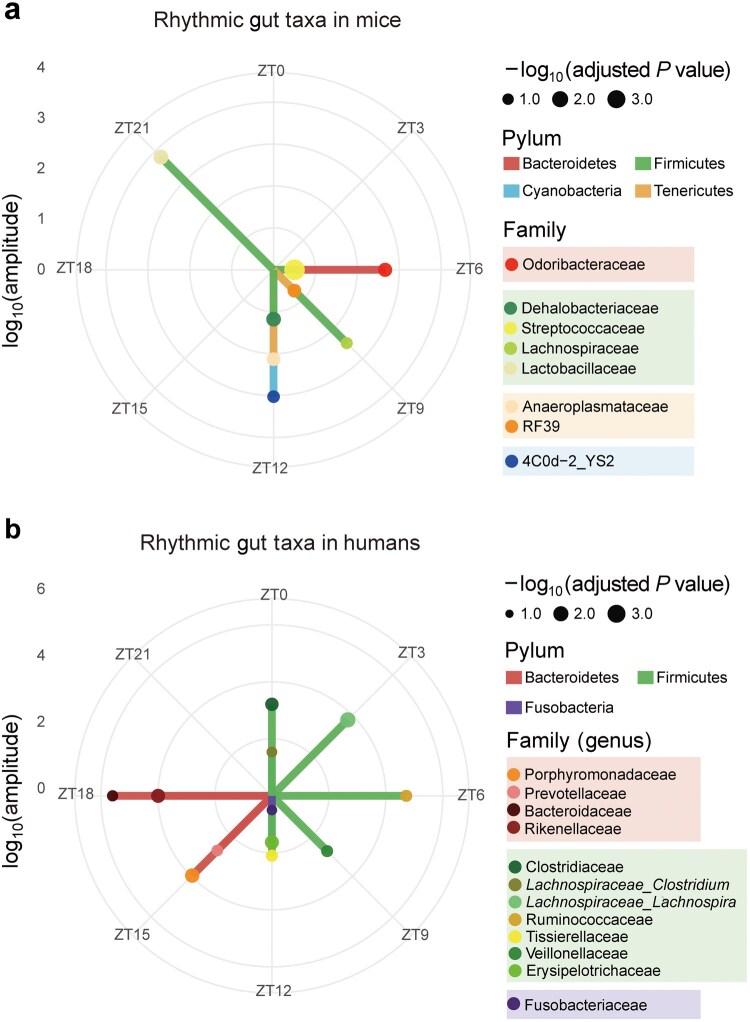
Diurnal phase distribution of rhythmic gut microbial taxa in mice and humans. Polar phase plots illustrate diurnally rhythmic gut microbial taxa in mice (a) and humans (b) using published 16S rRNA sequencing datasets analyzed with the JTK_CYCLE algorithm [69]. Only taxa exhibiting significant 24-h rhythmicity (adjusted *P *< 0.05) are included. Each point represents a rhythmic taxon, with angular position indicating the ZT of peak abundance derived from the JTK_CYCLE lag parameter. Radial distance corresponds to log_10_-transformed oscillation amplitude, reflecting the strength of diurnal fluctuation, while point size is proportional to rhythmic significance (−log_10_(adjusted *P* value)). Point colors denote taxonomic classification at the family level. In mice, 4C0d − 2_YS2 represents a combined class–order annotation, and RF39 denotes an order-level annotation for which family-level information is unavailable. In humans, *Lachnospiraceae_Clostridium* and *Lachnospiraceae_Lachnospira* indicate two genera within the Lachnospiraceae family that exhibit distinct peak abundances. Radial line segments connect each point to the center, with line colors indicating phylum-level classification.

The intestine represents the largest interface with the external environment, performing key physiological functions, including digestion, nutrient absorption, barrier defense, and immune regulation. The small intestine is primarily responsible for the digestion and absorption of nutrients and is characterized by its extensive array of villi and microvilli that maximize its surface area. The large intestine, in contrast, focuses on the reabsorption of water and electrolytes and the formation of feces. Both segments of the intestine exhibit a conserved multilayered structure typical of tubular organs, comprising from innermost to outermost, the lumen, mucosa, submucosa, and muscularis externa [9] (Fig. 2). The mucosal layer, located adjacent to the lumen, is further divided into the epithelium, lamina propria, and muscularis mucosae, serving as the primary site for nutrient uptake, immune surveillance, and mucosal motility.

The epithelial layer is continuously renewed by intestinal stem cells (ISCs), which differentiate into various specialized intestinal epithelial cells (IECs) (Fig. 2). Enterocytes are the most abundant IECs, featuring prominent brush borders and mediating the active absorption of carbohydrates, lipids, and amino acids [10]. Paneth cells, located at the crypt base, secrete antimicrobial peptides such as α-defensins and lysozyme, playing essential roles in microbial homeostasis and maintenance of the stem cell niche. Goblet cells produce mucins that form a protective mucus layer, preventing direct microbial contact with the epithelium. Tuft cells are rare chemosensory cells capable of detecting parasitic signals and initiating type 2 immune responses [11]. Enteroendocrine cells sense luminal nutrient content and release peptide hormones that regulate digestive and systemic metabolic functions. Additionally, microfold cells are specialized for antigen sampling and delivery to gut-associated lymphoid tissues, initiating mucosal immune responses. The lamina propria is densely populated with immune cells, including macrophages, dendritic cells (DCs), T cells, B cells, and plasma cells, forming the core effector population of the intestinal mucosal immune system [12]. These immune cells actively monitor luminal antigens and coordinate immune tolerance, pathogen clearance, and inflammatory control. Through this immunological infrastructure, the intestine not only provides a physical barrier but also serves as a central site for immune regulation and systemic homeostasis.

### Rhythmic renewal and differentiation of the intestinal cells

#### Rhythmic proliferation of IECs across organisms

The continuous renewal and orderly differentiation of IECs are essential for maintaining the gut barrier integrity and absorptive functions. Intestinal crypts, which are tubular glands located in the intestinal mucosa, form the niche for ISCs (Fig. 2). They provide both protection and key regulatory signals, serving as the starting point for epithelial regeneration. ISCs at the crypt base undergo self-renewal and differentiate into various specialized epithelial cells. These newly formed cells migrate upwards along the crypt-villus axis and are eventually shed at the villus tip, forming a complete cycle of approximately 3–7 days [13, 14]. In addition to this long-term renewal process, epithelial cell prolife­ration and differentiation are also tightly controlled by circadian rhythms within a 24-h cycle [15]. Direct *in vivo* evidence demonstrating causality between circadian clock activity in ISCs and defined functional outcomes such as barrier maintenance, nutrient absorption, or epithelial repair is still limited. However, growing evidence indicates that circadian regulation of ISC proliferation and differentiation could influence these intestinal functions [16]. This short-term regulation may help the intestinal epithelium respond to daily fluctuations in feeding, microbial exposure, and injury, suggesting a potential functional role that remains to be fully explored. Thus, ISC activity entrained by circadian signals may offer a new perspective for understanding multi-level temporal regulation within the epithelium.

Core clock genes play a critical role in regulating the circadian rhythm of ISC proliferation. A study using mouse intestinal organoids has revealed that Paneth cells, regulated by the core circadian transcription factor BMAL1, exhibit rhythmic secretion of Wnt ligands and serve to synchronize the proliferative activity of ISCs [17]. Another study found that in the mouse small intestine, crypt regeneration following γ-irradiation-induced damage exhibits a BMAL1-dependent circadian rhythm. Specifically, mitotic activity of injured ISCs peaks at ZT4, which is 4 h after light on, whereas the control uninjured crypts do not display significant circadian variation in mitosis. Furthermore, this rhythmic mitotic response is lost in *Bmal1* knockout mice, indicating that BMAL1 is essential for the temporal regulation of ISC proliferation during epithelial repair [18].

In zebrafish, ISC proliferation also shows diurnal variation. M-phase activity peaks at ZT21, while S-phase regulators such as proliferating cell nuclear antigen (PCNA) and cyclin-dependent kinase 2 (CDK2) reach their peak expression at ZT9. Although these genes do not respond directly to light–dark cycles, feeding time can reset the rhythm of *Per1*, thereby indirectly regulating cell cycle gene expression [19]. In *Drosophila*, ISC mitosis peaks at ZT0, while S-phase activity peaks at ZT6. These rhythms are lost in the *per* mutant, demonstrating the importance of circadian clock genes in maintaining cell cycle rhythmicity [20].

Human studies also support the existence of circadian regu­lation of intestinal epithelial proliferation. A biopsy study involving 23 subjects showed that epithelial cell proliferation in colonic crypts varies significantly throughout the day–night cycle, with a peak during the night and lower activity in the afternoon [21]. Another study found peak proliferation at 7:00 a.m., using 3H-thymidine labeling in rectal mucosa from 16 male volunteers [22]. The small peak proliferation difference between these studies may result from slight differences in biopsy locations, suggesting that cells at different positions along the crypt axis may have rhythms with slightly different phases.

#### The differentiation of intestinal cells is rhythmic

In addition to proliferation, circadian rhythms also influence epi­thelial cell differentiation. The number of tuft cells shows daily oscillation, regulated by histone deacetylase 3 (HDAC3), which promotes POU class 2 homeobox 3 (*Pou2f3*) gene expression through the transforming growth factor-beta/small mother against deca­pentaplegic (TGF-β/SMAD) pathway. This regulation controls the differentiation and abundance of tuft cells, and impacts the host’s susceptibility to norovirus infection in a time-of-day–dependent manner [11]. The number of goblet cells and their mucus secretion activity also show clear circadian variation. In rats, goblet cell abundance peaks at ZT21, with significantly lower counts during the daytime. This increase coincides with the onset of nocturnal feeding, a period associated with elevated exposure to food, gut microbes, and microbial metabolites. Increased mucus production can enhance mucosal barrier protection by limiting direct contact between luminal contents and the intestinal epithelium at this time [23]. In parallel, goblet cell secretion is higher during the active phase, supporting time-of-day regulation of epithelial defense [24]. Moreover, the enteric nervous system is also under circadian control. Enteric neural progenitor cells can differentiate into neurons and glial cells, and this process is influenced by circadian rhythms. Disruption of circadian rhythms by jet lag accelerates differentiation into glia and nitrergic neurons via the REV-ERBα (NR1D1)/nuclear factor-κB (NF-κB) axis, affecting gastrointestinal motility and function [25]. In summary, the proliferation and differentiation of intestinal epithelial and neural cells are under tight circadian regulation. These rhythms ensure the timely renewal of the gut barrier and coordinate mucosal functions with feeding and environmental cycles, thereby contributing to intestinal homeostasis and resilience to disease.

### Circadian rhythms of intestinal digestion and absorption

The intestine plays a central role in the digestion and absorption of nutrients such as carbohydrates, proteins, lipids, and micronutrients. Both the digestive enzyme activity and transporter gene expression are under circadian control, ensuring that nutrient breakdown and absorption are temporally aligned with the feeding schedule and environment light cycle.

#### Carbohydrate digestion and absorption

Disaccharidases, such as sucrase and maltase, are key enzymes that hydrolyze dietary disaccharides into monosaccharides for absorption. Absorption of dietary carbohydrates in the small intestine requires coordinated activity of disaccharidases and glucose transporters, primarily the sodium/glucose cotransporter 1 (SGLT1) and the facilitative transporter GLUT2. In rats maintained under a 12-h light/12-h dark cycle with *ad libitum* feeding, both sucrase activity and glucose absorption capacity exhibit circadian variation, peaking during the night, which corresponds to the active feeding phase of the animals [26–28]. Notably, the glucose absorption rhythm persists even under fasting conditions or altered light schedules, indicating that this rhythm is not only controlled by light or feeding cues, but also rhythmically regulated by transcription factors hepatocyte nuclear factor-1alpha (HNF-1α) and HNF-1β, histone acetylation, and the core clock proteins BMAL1 and PER1 [29–31]. Another study found that the activities of sucrase and maltase peak approximately 1 h before scheduled feeding and gradually decrease thereafter. Notably, this enzymatic rhythm persists for up to 48 h after food withdrawal and disappears after 5 days without feeding [32]. This anticipatory regulation indicates that digestive enzyme rhythms are entrained by habitual feeding schedules and are poised to peak in advance of food intake, ensuring that the digestive machinery is primed for optimal nutrient processing when feeding begins.

#### Amino acid digestion and absorption

Dipeptides and tripeptides derived from protein digestion are absorbed via peptide transporter 1 (PEPT1), encoded by *Slc15a1*, whose expression in mouse small intestine peaks around ZT12. This rhythm is regulated by the D-box binding PAR bZIP transcription factor (DBP), which binds to D-box motif sites in the PEPT1 promoter [33, 34]. Leucine aminopeptidase also contributes to protein digestion by cleaving amino acids from the N-terminus of peptide chains. In rats, the activity of leucine aminopeptidase in the small intestine exhibits pronounced circadian rhythms that are entrained by feeding time. Under *ad libitum* feeding, the enzyme activity peaks during the night and declines during the day, aligning with the natural nocturnal feeding behavior of the animals. However, when food is restricted to the light phase, the enzyme activity rhythms are reversed, with peak activity occurring during the day and reduced activity at night [28].

#### Lipid digestion and absorption

Proteins involved in lipid absorption such as microsomal triglyce­ride transfer protein (MTP) and apolipoprotein A-IV (ApoA-IV) exhibit robust daily rhythms. In mice, MTP and ApoA-IV expression peaks at ZT12, promoting chylomicron assembly and triglyceride transport during the nocturnal feeding period [35]. In rats, both triglyceride and cholesterol absorption are highest during the night, which matches the peak of MTP activity. Constant light or dark conditions disrupt these rhythms, and Clock-mutant mice show lipid malabsorption and hyperlipidemia due to dysregulated transporter expression [36, 37]. Also, in IECs, both the transcription of *CD36*, which facilitates dietary fat absorption, and that of fatty acid binding protein 4 (*FABP4*), which mediates intracellular fatty acid transport, storage, and signaling, peak at ZT14. Carnitine palmitoyltransferase 1A (CPT1A), which is involved in fatty acid β-oxidation and lipid biosynthesis, reaches a nadir at ZT8. Together, these studies reveal clear circadian rhythmicity in lipid metabolic pathways [38, 39].

#### Micronutrient absorption

Both iron and calcium absorption in the small intestine are subjected to circadian regulation. The expression of iron uptake and transporter genes, including divalent metal transporter 1 (*DMT1*), cytochrome B reductase 1 (*CYBRD1*), and ferroportin (*FPN*), exhibits diurnal variation entrained by feeding time, with enhanced iron storage and gene expression when iron is administered in the evening. This is accompanied by elevated expression of clock genes such as *Per2*, *Cry1*, and *Cry2* [40]. In parallel, calcium absorption is regulated post-translationally via the circadian expression of ubiquitin-specific protease 2-45 (USP2-45), which modulates the membrane protein NHERF4, a key regulator of the TRPV6 calcium channel. Disruption of core clock genes *Cry1* and *Cry2* abolishes this rhythmic regulation, highlighting the essential role of the intestinal circadian system in coordinating mineral metabolism [41]. Together, these findings demonstrate that the intestinal circadian clock fine-tunes mineral absorption to align with daily nutritional demands.

#### Influence of peripheral clocks, feeding time, and light on digestion and absorption

Feeding behavior itself serves as a powerful zeitgeber for entraining the intestinal clock. Restricted feeding studies in nocturnal rodents have shown that shifting food availability to the rest phase (i.e. light period) inverts the diurnal rhythms of nutrient transporter transcripts, such as *Sglt1* and *Pept1*, in the duodenum, demonstrating that feeding time alone can reprogram intestinal circadian activity independent of the light–dark cycle [42].

Light is the primary synchronizer of the central clock in the SCN of the hypothalamus. The SCN synchronizes peripheral tissues through neuroendocrine signaling pathways, including rhythmic secretion of glucocorticoids and melatonin. Disruption of light cues leads to desynchronization between the SCN and intestinal clocks, such as that caused by constant light or darkness. This causes downstream hormonal rhythms to become mistimed, leading to peripheral organs, such as the intestine, losing the external signals which they rely on to maintain daily oscillations. As a result, digestive enzyme expression and nutrient transporter activity drift out of phase or become arrhythmic, reducing their ability to match the timing of feeding and thereby impairing digestive efficiency [36].

Sugar-digesting enzymes such as sucrase and maltase ­exhibit activity rhythms that are tightly linked to scheduled feeding. Notably, their enzymatic activities begin to rise about 1 h before feeding and decline afterward. This anticipatory activation persists even in the absence of food for up to 2 days, suggesting that in addition to actual ingestion, anticipatory feeding in rats can also influence digestive enzyme rhythms in the gut [32]. This regulation may reflect gut intrinsic circadian rhythms or be secondary to feeding patterns driven by central circadian regulation.

In summary, the circadian control of intestinal digestion and absorption is the result of an integrated system in which feeding schedules entrain local intestinal clocks, while light cues regulate systemic synchrony via the SCN. Disruption either of feeding time or light exposure can disturb the coordination of nutrient breakdown and uptake, ultimately affecting gut function and whole-body metabolic homeostasis.

### Circadian regulation of gut hormones

Beyond its function in nutrient absorption, the intestine is a highly active endocrine organ that secretes a wide array of peptide hormones. These gut-derived hormones play essential roles in regu­lating appetite, glucose metabolism, gastrointestinal motility, and overall energy balance. Importantly, the production and secretion of these hormones exhibit robust circadian rhythms that align with feeding behavior and energy demand. This temporal regulation allows the body to anticipate nutrient intake and orchestrates hormonal signaling for optimal metabolic control.

Among the most studied gut hormones with circadian regulation are glucagon-like peptide-1 (GLP-1), glucose-dependent insulinotropic polypeptide (GIP), and peptide YY (PYY) [43–45]. These hormones are secreted by specialized enteroendocrine cells located within the intestinal epithelium. Specifically, K cells, a subset of enteroendocrine cells located predominantly in the duodenum and jejunum, secrete GIP in response to the presence of nutrients. In contrast, L cells, distributed throughout the distal small intestine and colon, secrete both GLP-1 and PYY [46]. These cells act as nutrient sensors, rapidly responding to carbohydrates, lipids, and amino acids in the gut lumen by releasing hormones into the circulation. Their activities are tightly regulated by feeding cues and intrinsic molecular clocks, enabling time-of-a-day–specific hormonal responses.

GLP-1 and GIP are classic incretin hormones that enhance glucose-dependent insulin secretion from pancreatic β-cells and delay gastric emptying. In healthy humans, both hormones show clear diurnal fluctuations, peaking during daytime feeding pe­riods, particularly around breakfast and lunch. This anticipatory secretion is aligned with meal timing to support postprandial glucose control. However, in individuals with obesity, the amplitude of these hormonal rhythms is significantly reduced and remains disrupted even after weight loss, indicating a persistent impairment in circadian regulation [44]. Experimental studies in rodents have shown that GLP-1 secretion begins before the expected feeding period, peaking at ZT6–ZT10 and reaching a nadir at ZT22. Under conditions of constant light exposure, the rhythmic secretion of GLP-1 and insulin is abolished, and both hormones remain elevated throughout the 24-h cycle [43]. This indicates that L cell rhythms are not solely entrained by food but also dependent on intact light–dark cues.

PYY is also secreted by L cells, reducing upper gastrointestinal motility and prolonging nutrient contact time in the intestine. In humans, PYY levels peak after lunch, reflecting a postprandial response tied to meal timing [47]. PYY is closely associated with eating rhythms; not only does eating modulate its secretory rhythm, but the satiety provided by PYY can in turn influence feeding rhythms as well [48].

In addition to these core hormones, oxyntomodulin (OXM) and neurotensin (NT), which are co-secreted with GLP-1 and PYY, also display circadian rhythmicity. OXM is released by L cells and functions via dual activation of the GLP-1 and glucagon receptors to reduce appetite and increase energy expenditure. Its secretion peaks during the active/feeding phase and is abolished by constant light [49]. NT, produced by N cells in the distal small intestine, modulates lipid absorption and intestinal repair. In the rodent model, NT levels peak in the early morning (ZT0–ZT4) and decrease through the day, a pattern that persists under light–dark cycles but not under constant illumination [50]. NT is co-expressed with GLP-1 and PYY, and acts synergistically to regulate energy balance and gut motility.

Insulin is a key anabolic hormone secreted by pancreatic β-cells in response to elevated blood glucose levels. It facilitates glucose uptake, suppresses hepatic glucose production, and regulates lipid and protein metabolism, thereby playing a central role in maintaining glucose homeostasis. Importantly, insulin secretion follows a circadian rhythm, with peak levels typically occurring during the active or feeding phase in both human and rodents [51, 52]. This rhythm is strongly influenced by feeding behavior; restricted feeding schedules can shift the timing of insulin secretion, and food intake during the rest phase leads to exaggerated insulin responses compared to feeding during the active phase [53]. Recent findings suggest that these feeding-related changes in insulin rhythmicity may be mediated through the circadian rhythms of the gastrointestinal tract. Among these, gut hormones such as GIP and GLP-1 show pronounced circadian secretion and act as potent enhancers of glucose-stimulated insulin release [44]. Therefore, it is plausible that feeding-induced gut rhythms, together with central clock signals, act through the rhythmic release of gut hormones and nutrient signals, which aligns insulin secretion with external feeding cues.

In summary, the circadian rhythms of intestinal hormones are regulated at both the molecular and behavioral levels. Core hormones such as GLP-1, GIP, PYY, OXM, and NT exhibit time-of-day–dependent fluctuations driven by intrinsic clocks in enteroendocrine cells and shaped by external cues such as feeding schedule and light exposure. For insulin, although there is no direct evidence that gut circadian rhythms alone drive its oscillations, the rhythmic release of gut hormone such as GLP-1 and GIP suggests that they may serve as intermediaries linking the gut clock to the insulin circadian pattern. This rhythmic regulation ensures that nutrient sensing, glucose handling, and satiety signaling are aligned with feeding activities. Hormones also serve as a key bridge through which the gut influences systemic physiology and phenotype.

### Circadian regulation of intestinal motility

Intestinal motility is a fundamental physiological function that supports food digestion, nutrient absorption, and waste elimination. It is an active process involving coordinated contractions of smooth muscle, input from enteric nervous system, and hormonal and central nervous system. Increasing evidence shows that intestinal motility exhibits robust circadian rhythms that are not solely dependent on feeding but are also regulated by endogenous molecular clocks [54].

In humans, colonic motility is not strongly influenced by the light cycle. It is primarily regulated by the sleep–wake cycle, with motility enhanced during waking hours and postprandial periods, but markedly reduced during sleep [55]. In rotating shift workers, this rhythmic pattern is disrupted. Such circadian misalignment leads to blunted colonic motor activity, reduced defecation frequency, and increased rectal compliance. These factors impair visceral sensory thresholds and modulate anorectal neuromuscular control [56]. Rodent models offer complementary evidence for intrinsic circadian regulation of intestinal motility. In mice, parameters such as colonic pressure waves, fecal pellet output, and smooth muscle contractility follow clear daily rhythms: colonic tone and fecal output peak during the nocturnal active phase and decline during the daytime rest phase. In contrast, *Per1*/*Per2* double knockout mice lose these rhythmic patterns, indicating that core clock genes drive the rhythmic control of gut motility [57].

In summary, intestinal motility follows a robust circadian pattern that aligns gastrointestinal activity with behavioral states such as wakefulness and feeding. These rhythms are regulated by intrinsic molecular clocks and reinforced by systemic cues. Disruption of these rhythms can impair colonic function, reduce motility, and potentially contribute to gastrointestinal dysfunction due to genetic mutation or circadian misalignment, such as night shift work.

### Circadian regulation of gut immune function

The gastrointestinal tract is not only responsible for digestion and nutrient absorption but also functions as the largest immune organ of the body. Constantly exposed to food antigens, commensal organisms, and potential pathogens, the gut must maintain a fine balance between immune tolerance and defense. This is achieved through a multilayered immune system consisting of a physical epithelial barrier, innate immune cells that provide rapid responses, and adaptive immune cells that mediate specific recog­nition and memory. Growing evidence shows that gut immune functions are regulated by circadian rhythms that align immune readiness with feeding behavior, microbial exposure, and tissue renewal. Understanding how these immune rhythms are orga­nized provides critical insights into gut defense mechanisms and how disruptions in timing may lead to inflammation, infection, or immune-mediated diseases.

At the first level of defense, circadian rhythms help maintain gut immune homeostasis by coordinating barrier function and immune responsiveness. The intestinal epithelial barrier is dynamically regulated across the day. Key tight junction proteins, including occludin, claudin-1, and zonula occludens-1 (ZO-1), exhibit peak expression during the dark phase around ZT12–ZT16 in nocturnal rodents, coinciding with highest luminal antigen exposure, thereby enhancing barrier integrity and reducing permeability [58]. Furthermore, chromatin-modifying enzymes are also involved in this regulation; for instance, HDAC3 acts as a negative regulator of claudins, and loss of HDAC3 in mouse IECs attenuates the rhythmic histone 3 lysine 9 acetylation (H3K9ac) and histone H3 lysine 27 acetylation (H3K27ac) signals at the claudin promoters [59].

Beyond the epithelial barrier, the innate immune system shows pronounced circadian patterns. Macrophages and group 3 innate lymphoid cells (ILC3s) are notable examples. In peritoneal macrophages, BMAL1 controls rhythmic expression of pro-inflammatory cytokines, with tumor necrosis factor-α (TNF-α) peaking at ZT16 and interleukin-6 (IL-6) peaking at ZT8 in response to lipopolysaccharide stimulation [60]. In the intestine, ILC3s oscillate, with cell numbers peaking at ZT12 in the lamina propria. Importantly, one of their key effector cytokines, IL-22, which promotes antimicrobial peptide production and barrier integrity, also exhibits diurnal oscillations, peaking at ZT6. Disruption of BMAL1 in ILC3s abolishes IL-22 rhythmicity and worsens inflammation [61]. Another critical innate immune component is immunoglobu­lin A (IgA), which regulates the composition and metabolic ­activity of the commensal microbiota. Intestinal IgA secretion shows robust diurnal rhythmicity, peaking around ZT6 and reaching a nadir around ZT18 in both fecal samples and small intestinal IgA^+^ plasma cells. Transcriptomic profiling revealed that approximately 16% of genes expressed in these plasma cells oscillate across the day, including core clock genes *Bmal1* and *Per2* [62, 63].

Finally, the adaptive immune system in the gut is also under circadian control, with T and B lymphocyte subsets showing time-of-day–dependent activity. For example, CD4^+^ T cells in gut-associated lymphoid tissues exhibit rhythmic migration and cytokine production, with T helper type 1 (Th1) cells reaching peak interferon gamma (IFN-γ) production around ZT12–ZT16 under PER2 regulation [64]. Th17 cells in the small intestine fluctuate with a peak at ZT18, dependent on T cell-intrinsic *Bmal1* [65]. Regulatory B cells also follow a daily rhythm, which is partly driven by IL-33; IL-33 expression peaks at ZT16 in wild-type mice but is lost in *Bmal1*-deficient mice [66]. Other adaptive immune effectors also follow circadian patterns: antimicrobial peptides such as regenerating islet derived protein 3 gamma (Reg3γ) peak around ZT12, while major histocompatibility complex (MHC) class II molecules, which is critical for antigen presentation to CD4^+^ T cells, exhibit BMAL1-dependent rhythmicity with maximal expression at ZT12 [67, 68].

Taken together, circadian rhythms orchestrate intestinal immunity across three interconnected layers: strengthening the epi­thelial barrier during periods of maximal exposure, modulating innate immune activity through transcriptional clock programs, and temporally aligning adaptive responses and antibody secretion. This time-based organization enables the gut to anticipate environmental challenges and defend efficiently, while minimizing unnecessary inflammation.

## Gut microbiota and circadian rhythms

The gut microbiota is a highly dynamic ecosystem that interacts intimately with the host circadian system. Mounting evidence reveals that microbial communities exhibit robust daily oscillations in abundance, transcriptional activity, and metabolite production, and that these rhythms are temporally aligned with the circadian clock of the host. This bidirectional relationship is mediated through multiple pathways, including diet, host-derived hormones, and microbial metabolites, which form multiple overlapping feedback loops. These loops, including those between feeding rhythms and microbial metabolism, circadian hormones and microbial activity, and immune signaling and microbial composition, collectively synchronize microbial activity with host metabolism, immunity, and neural function. Disruption of these rhythms can lead to microbiota imbalance and contribute to disorders ranging from metabolic disease to immune dysfunction due to clock gene mutations or lifestyle misalignment, such as irregular feeding and jet lag. In this chapter, we review the circadian rhythms in the gut microbiota, how microbial metabolites convey rhythmic signals to the host, and how the host circadian machinery shapes microbial structure and function, ending in a discussion of their health implications and therapeutic potential.

### Rhythms of gut microbial abundance and composition

Many studies have shown that the gut microbiota is not constant, but changes in a regular daily rhythm. In mice, a substantial proportion of bacterial taxa show daily fluctuations in their relative abundance. For example, more than 15% of operational taxonomic units (OTUs) in fecal samples exhibit robust 24-h rhythmicity (Fig. 2a). These rhythmic bacteria include *Lactobacillus reuteri*, *Dehalobacterium* spp., and multiple species within the orders Clostridiales, Lactobacillales, and Bacteroidales [69]. These microbes are mostly commensal or mutualistic and play critical roles in host physiology. *L. reuteri* is a well-known probio­tic that promotes gut barrier function and immune modulation; Clostridiales includes butyrate-producing species important for colon health; and Bacteroidales members are major contributors to carbohydrate fermentation and immune education.

The timing of these oscillations is also closely aligned with host feeding behavior. In fecal pellets from C57BL/6 mice, total bacterial biomass is significantly higher during the dark/feeding phase in mice [70]. This suggests that nutrient availability is a key driver of microbial growth and rhythmicity. Functionally, microbial gene expression also oscillates in time with host activity. Genes related to energy metabolism, DNA repair, and cell division peak during the active/dark phase, while those involved in detoxification and environmental sensing are more abundant during the rest/light phase [69]. This rhythmic expression may allow microbes to anticipate changes in their environment and optimize resource use.

In humans, microbial rhythms still exist, but their patterns can vary and become less pronounced due to differences in lifestyle, diet, and broader ranges of daily activity. Nonetheless, roughly 10% of microbial taxa and about 20% of metabolic pathways in human gut microbiome exhibit daily rhythmicity [69] (Fig. 2b). *Parabacteroides*, *Lachnospira*, and *Bulleidia* have been found to have robust oscillations. Among these microbes, *Roseburia* is a key producer of short-chain fatty acids (SCFAs), which have important roles in the gut.

*Akkermansia muciniphila* is a key mucin-degrading bacterium in human gut that plays an important role in maintaining metabolic health, improving gut barrier integrity, and modulating host immune responses. Studies have shown that administration of live or pasteurized *A. muciniphila* reduced fat mass, improved insulin sensitivity, and modulated energy absorption, making it a promising target for metabolic disorders such as obesity and type 2 diabetes (T2D) [71]. Notably, the abundance of *A. muciniphila* exhibits diurnal oscillations that aligns with host feeding rhythms, fluctuating in synchrony with bile acid profiles and lipid metabolism, indicating an intrinsic rhythmicity that can be disrupted by mistimed feeding [72].

**Figure 3:**
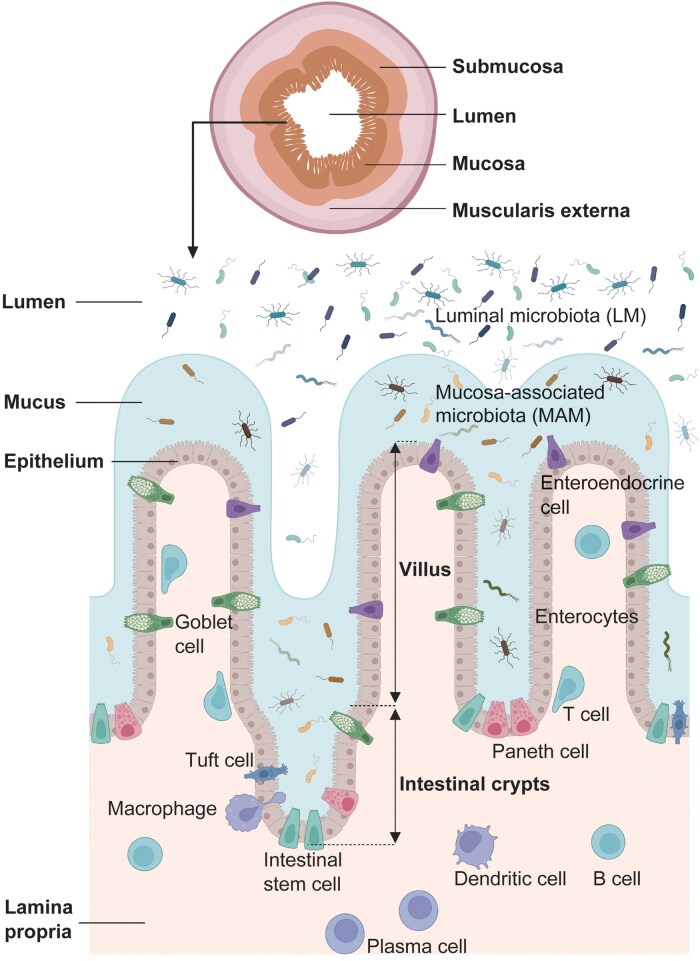
Structural and cellular organization of the intestinal mucosa layer. This figure illustrates the cross-sectional anatomy and cellular composition of the intestinal mucosa. On the top, the layered structure of the gut wall is shown, including the lumen, mucosa, submucosa, and muscularis externa. The bottom panel provides a detailed view of the intestinal epithelium and underlying lamina propria, highlighting key epithelial cells (e.g. enterocytes, stem cells, goblet cells, Paneth cells, tuft cells, and enteroendocrine cells) and immune cells (e.g. macrophages, DCs, T cells, B cells, and plasma cells). The figure also delineates LM and MAM, emphasizing the spatial organization of microbes relative to the mucus layer and host cells.

In addition, the spatial localization within the gut may contri­bute to region-specific rhythmic patterns among gut microbes. The gut microbiota can be broadly categorized into two groups based on their location: luminal microbiota (LM) and mucosa-associated microbiota (MAM). LM are free-floating microbes found in the gut lumen and are typically detected in fecal and intestinal content samples (Fig. 3). They play a major role in fermenting dietary fiber and other complex carbohydrates, producing SCFAs and other beneficial metabolites [73]. In contrast, MAM are primarily located in the mucus layer near the intestinal epithelium. MAM are crucial for the development and maturation of the host immune system. They interact directly with epithelial cells and help maintain gut barrier integrity by influencing immune responses and promoting the secretion of mucus and antimicrobial compounds [74]. LM are considered more sensitive to dietary changes, while MAM tend to be more stable and less affected by short-term dietary variations [75]. Therefore, even within the same gut region, the rhythmic patterns of LM and MAM may differ [76]. However, current mainstream methods for studying gut microbial rhythms mainly rely on metagenomic and 16S sequencing of fecal or luminal contents. The circadian rhythms of mucosa-associated microbes remain relatively unexplored and may represent a new and valuable research direction.

Altogether, gut microbial rhythmicity spans diverse bacterial taxa with important physiological roles. These rhythms are synchronized with host behaviors, particularly feeding, and represent a coordinated adaptation to daily rhythmic environment of the host. Understanding the identities, timing, and spatial dynamics of rhythmic microbes lays the groundwork for deciphering how the microbiome contributes to systemic host rhythms and health.

### Rhythms of microbial metabolites

In addition to the rhythms of microbial abundance and composition, circadian rhythms are increasingly observed in microbial metabolic activities. Recent studies demonstrate that gut microbial metabolites, including SCFAs, bile acids, tryptophan derivatives, and neurotransmitters such as γ-aminobutyric acid (GABA) and 5-hydroxytryptamine (5-HT), also exhibit significant diurnal fluctuations. These metabolites can actively modulate host metabolic, immune, and neurological functions.

#### SCFAs

**Figure 4:**
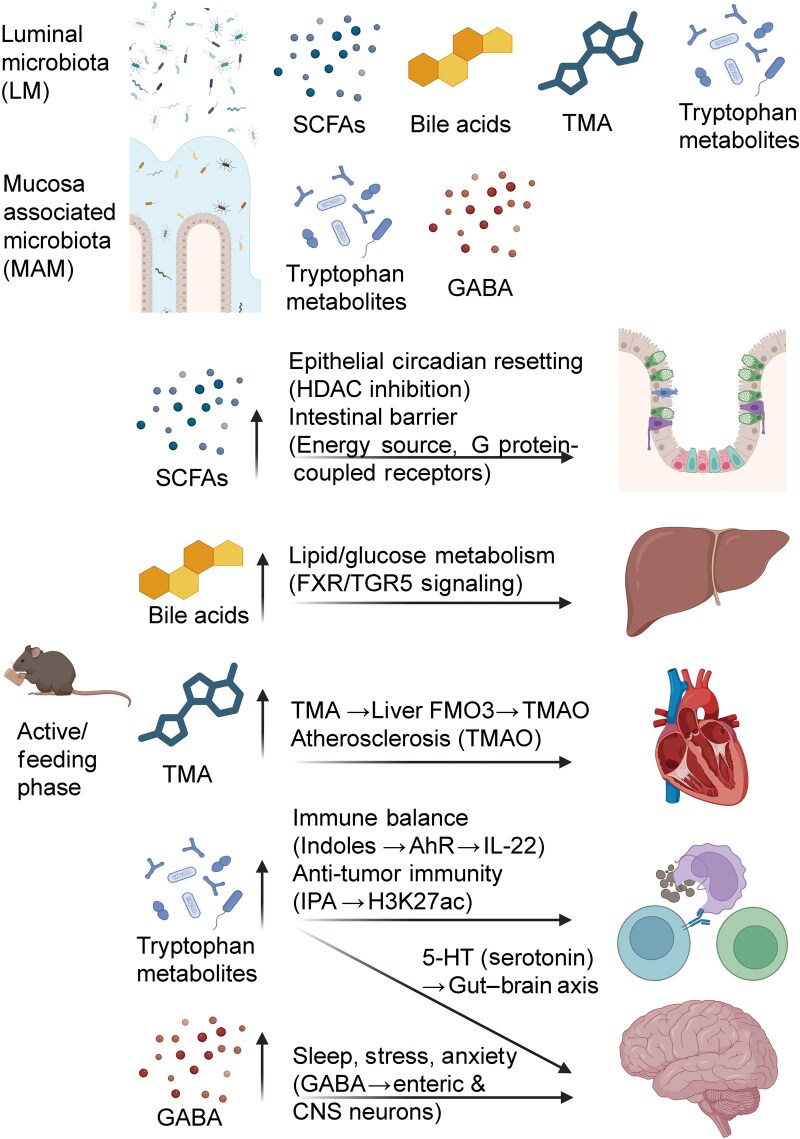
Microbial metabolites and host circadian regulation. LM mainly produce SCFAs, bile acids, tryptophan derivatives, and TMA, while MAM contribute to GABA and mucosal tryptophan metabolism. These metabolites act on host pathways in a rhythmic manner: SCFAs reset epithelial clocks and strengthen barrier function; bile acids regulate lipid/glucose metabolism and circadian genes via FXR/TGR5; TMA is converted by hepatic FMO3 to TMAO, promoting atherosclerosis; tryptophan metabolites modulate immunity (AhR and IL-22) and anti-tumor responses, while serotonin links to the gut–brain axis; and GABA influences sleep and stress resilience through enteric and central neurons.

SCFAs, mainly acetate, propionate, and butyrate, are fermentation products of dietary fiber and exhibit robust daily oscillations in the gut. Their levels rise during the active/feeding phase and decline during the resting phase, reflecting the interplay between feeding schedules and microbial metabolism [77]. SCFAs regulate host physiology through several mechanisms. They inhibit HDACs and thereby reshape gene expression, activate G protein-coupled receptors such as GPR41, GPR43, and GPR109A to trigger signaling cascades [78, 79] and provide energy for enterocytes, supporting proliferation, differentiation, and barrier integrity [80, 81] (Fig. 4). For instance, butyrate promotes regulatory T cell (Treg) differentiation by enhancing histone H3 acetylation at the *Foxp3* locus [82], while GPR41 activation can induce sympathetic nervous activity [83]. Notably, SCFAs also act as zeitgebers for peripheral clocks. By modulating HDAC activity, they induce phase shifts in circadian gene expression in the intestine and other tissues [5, 84]. Dietary interventions can alter SCFA rhythms: for instance, oat supplementation raises gut SCFA levels and in turn influences hepatic circadian programs [85].

#### Bile acids

Bile acids, crucial for lipid emulsification and absorption, also act as signaling molecules (Fig. 4). Their rhythmic transformation is mediated by gut microbial enzymes such as bile salt hydrolase (BSH) and dehydroxylase, which regulate bile acid diversity and function [86]. Primary bile acids such as cholic acid are secreted during the day and transformed into secondary bile acids such as deoxycholic acid and lithocholic acid at night [87]. These metabolites act through farnesoid X receptor (FXR) and G protein-coupled bile acid receptor (TGR5, also known as GPBAR1) to regulate lipid and glucose metabolism and inflammation. Disruption of bile acid rhythmicity contributes to metabolic syndromes, fatty liver, and insulin resistance [88, 89].

Moreover, the hepatic gene cytochrome P450 family 7 subfami­ly A member 1 (*Cyp7a1*), which encodes the enzyme cholesterol 7α-hydroxylase responsible for bile acid synthesis, is under circadian regulation. In wild-type mice, *Cyp7a1* mRNA exhibits strong circadian oscillation, while in Clock mutant mice, the rhythm is dampened. These findings suggest that the circadian regulation of bile acid synthesis depends on the coordinated actions of clock transcription factors [90]. Importantly, unconjugated bile acids, produced by microbial BSH activity, have been shown to enhance the expression of core clock genes such as *Clock* and *Bmal1*, and their oral administration modifies clock gene expression in the ileum, colon, and liver, including *Dbp*, *Per2*, *Per3*, and *Cry2* [91]. These effects suggest that bile acids generated by gut microbes may act as diurnal entraining signals for peripheral clocks, particularly in the intestinal epithelium.

Together, these data emphasize that gut microbial regulation of bile acid composition and timing is not only central to metabolic health but also serves as a mechanism by which the microbiome entrains host circadian rhythms.

#### Tryptophan metabolites

Tryptophan, an essential aromatic amino acid, serves as a meta­bolic hub linking dietary inputs, microbial activity, and host physiology through three principal pathways: the serotonin (5-HT) pathway, the kynurenine pathway, and the indole pathway (Fig. 4). Among these, the indole pathway is exclusively microbially mediated, producing a range of bioactive metabolites, such as indole-3-lactic acid (ILA), indole-3-propionic acid (IPA), indole-3-acetic acid (IAA), and indole-3-aldehyde (IAld), many of which act as ligands for the aryl hydrocarbon receptor (AhR). These microbial metabolites exhibit pronounced circa­dian rhythmicity in both production and function. In conventional mice, approximately 60% of rhythmic gut microbes carry tryptophan-metabolizing genes [92]. Among these, the pathway for tryptophan degradation is more active during the inactive (light) phase, whereas kynurenine degradation, a downstream branch of tryptophan degradation, shows oscillatory rhythmicity with peak activity during the active (dark) phase. Several predicted tryptophan-related metabolites, including serotonin, melatonin, kynurenine, and indole derivatives, also have circadian rhythms. Tryptophan metabolic genes, including tryptophan hydroxylase 1 (*Tph1*) and indoleamine 2,3-dioxygenase 1 (*Ido1*), as well as the receptors AhR and pregnane X receptor (PXR), exhibit significant diurnal variation in both the ileum and the colon [92]. Germ-free mice or antibiotics-treated mice show dampened or abolished rhythms, especially in AhR-related signaling genes such as cytochrome P450, family 1, subfamily A, polypeptide 1 (*Cyp1a1*), aryl hydrocarbon receptor repressor (*Ahrr*), and *Pxr*. Functionally, tryptophan metabolites exert time-dependent effects on host physiology. For example, IPA enhances antitumor immunity by epigenetically reprogramming TCF1^+^ progenitor exhausted CD8^+^ T cells via histone H3K27 acetylation, thereby improving the efficacy of the programmed cell death protein 1 (PD-1) checkpoint blockade therapy [93]. Similarly, ILA and IAld produced by *Lactobacillus* species activate AhR in a context-dependent manner to regulate immune responses and barrier integrity, with peak activity aligned to nocturnal synthesis phases. Importantly, rhythmic tryptophan metabolism not only affects local gut physiology but also modulates circadian clock outputs in other tissues, such as SCN and liver transcriptional programs, demonstrating that tryptophan acts as a zeitgeber buffer linking dietary cues to circadian homeostasis [94–96]. Together, these findings position microbial tryptophan metabolism as a dynamic, time-regulated interface critical for host-microbe circadian synchrony and metabolic health.

#### GABA

The gut microbiota also contributes to circadian regulation of neural activity through the production of GABA, the principal inhibitory neurotransmitter. In addition to being synthesized in the brain, GABA is also produced by intestinal microbes such as *Lactobacillus* and *Bifidobacterium* species. Studies have shown that germ-free mice have significantly lower GABA levels in both feces and circulating blood compared to conventionally raised mice, highlighting the role of the microbiota in peripheral GABA synthesis and its potential influence on host neurophysiology. Disruption of microbial GABA production via antibiotics, dietary changes, or circadian rhythm interruption has been associated with increased anxiety-like behavior, impaired sleep, and reduced stress resilience [97]. Furthermore, modifying the gut microbiota with antibiotics such as neomycin significantly increases fecal GABA levels without affecting serum concentrations, suggesting enhanced localized production within the gut epithelium [98].

Overall, microbial modulation of tryptophan metabolism and GABA production links intestinal microbes to the neural activity and circadian behavior of the host. These microbial metabolites synchronize peripheral neural and immune responses with the central circadian clock, potentially influencing mood, cognition, and sleep–wake cycles [99].

#### Trimethylamine (TMA) and trimethylamine N-oxide (TMAO)

TMA is made by gut microbes from foods rich in choline and L-carnitine, and is then converted in the liver by the enzyme flavin-containing monooxygenase 3 (FMO3) into TMAO. High levels of TMAO are linked to heart and metabolic diseases, including atherosclerosis and insulin resistance. In mice, the liver enzyme FMO3 shows clear daily rhythms, and microbial production of TMA plays an important role in setting host circadian rhythms [100, 101]. When either the microbial genes that make TMA or the host enzyme that converts TMA to TMAO is removed, the normal circadian rhythm of the host is strongly disrupted. The levels of metabolites in this pathway also fluctuate diurnally: choline, L-carnitine, and TMA are lower during the light (resting) phase and peak around ZT12, when mice begin to eat [102]. In addition, inhibiting the gut microbial enzyme choline TMA-lyase (CutC), which drives TMA/TMAO production, protects against obesity-related metabolic dysfunction by reshaping the microbiome, improving glucose metabolism, and reorganizing host circadian control of energy balance [103].

### Host regulation of microbial rhythms

The rhythmic behavior of gut microbes is shaped by the intrinsic circadian clock of the host. Evidence from clock gene knockout studies in mice shows that disruption of key circadian regulators in the host leads to a loss of gut microbial rhythmicity. For instance, compared to wild-type mice, *Per1/2^–/–^* mice show a nearly complete loss of rhythmic fluctuations in the abundance of commensal bacteria. The number of bacterial taxa with diurnal oscillations is greatly reduced, and the few taxa that retain rhythmicity display almost random changes in abundance [69]. In addition, the rhythmic functions of these gut microbes, such as vitamin and nucleotide metabolism, secretion systems, DNA repair, cell wall synthesis, and motility, lose their diurnal rhythmicity in *Per1/2^–/–^* mice. Likewise, deletion of *Bmal1* also abolishes the rhythmicity of the gut microbiota in mice, as shown by the absence of temporal changes in microbial abundance over the 24-h cycle [70]. Notably, these effects are not secondary to general behavior or metabolic disturbances, as *Bmal1* deletion restricted to IECs still results in loss of microbial rhythmicity. This suggests that peripheral clocks within the gut epithelium are sufficient to impose rhythmic constraints on the microbial ecosystem.

The host also exerts control of the gut microbiota through rhythmic secretion of hormones. Melatonin, a hormone with well-established circadian expression, was shown to enhance rhythmic behaviors in *Enterobacter aerogenes*, including swarming and motility, under physiological concentrations *in vitro* [104]. These findings suggest that some gut bacteria may directly respond to host-derived hormonal cues. In addition, diurnal fluctuations of hormones such as melatonin and cortisol in the host also affect gastric acid secretion, bile acid metabolism, and gastrointestinal peristalsis, thereby indirectly regulating the growth of gut microorganisms [105, 106].

Sex-based differences in microbial rhythms have also been reported. Both male and female mice exhibit daily oscillations of the gut microbiota, but females tend to show higher amplitude in daily microbial shifts. However, disruption of the *Bmal1* gene eli­minates these sexual differences, highlighting the dominant role of the host circadian clock in shaping microbial rhythmicity [70]. This observation suggests that while sex may modulate rhythmic amplitude, a functional molecular clock is essential for establishing microbial periodicity.

Together, these studies highlight the role of host circadian system in shaping the rhythmic behavior of gut microbes. Disruption of core clock genes like *Per1/2* or *Bmal1* abolishes microbial rhythmicity, even when the perturbation is restricted to gut epithelial cells, indicating the importance of local peripheral clocks. Additionally, while sex-based differences in microbial rhythms exist, they are abolished in the absence of a functional circadian clock, underscoring an important role of host circadian system regulating microbial oscillations.

### Environmental factors influencing microbial rhythms

While host genetics provides the foundation for microbial rhythmicity, environmental inputs—particularly those related to light exposure, feeding behavior, and diet—also serve as powerful modulators of microbial oscillations in the gut. Experimental studies in mice typically use a controlled 12-h light/12-h dark cycle (LD 12:12) with *ad libitum* feeding to assess normal microbial rhythmicity. Under these conditions, gut bacteria display clear daily fluctuations in both abundance and function, tightly synchronized with the feeding and activity cycles of the host.

#### Light–dark and sleep schedule disruption perturbs gut microbial circadian rhythms

Chronic circadian disruption, such as jet lag or rotating light–dark schedules, markedly impairs the composition and rhythmicity of the gut microbiota. In mouse models exposed to repeated 12-h light/12-h dark phase shifts, mimicking transmeridian travel, microbial diversity is reduced, and the temporal structure of the gut microbiome becomes disorganized, leading to a loss of rhythmic taxa such as *Lactobacillus* and members of the Ruminococcaceae family [107]. These disruptions are associated with increased intestinal permeability, endotoxemia, and inflammation, likely mediated by altered expression of tight junction proteins such as occludin. Importantly, fecal microbiota transplantation (FMT) experiments demonstrate that circadian-disrupted microbiota can transfer metabolic dysfunction to germ-free recipients. For instance, microbiota from jet-lagged mice, when transplanted into germ-free hosts, impairs glucose tolerance and disrupts recipient microbial rhythmicity, indicating that the adverse metabolic effects of circadian misalignment are at least partly mediated through the microbiome. Similarly, FMT from mice housed under rotating light–dark cycles results in dampened diurnal oscillations of bacterial taxa in recipient mice, reinforcing the concept that the gut microbiota can transmit circadian misalignment to host physiology [108]. Moreover, sleep timing affects the gut microbiota, including the abundance of *A. muciniphila* in children, implying that healthy sleep habits may indirectly support metabolic benefits mediated by *Akkermansia* [109]. These findings highlight that jet lag not only disturbs microbial rhythmicity but also generates a dysbiosis state that is transmissible and can independently impair host metabolic and immune homeostasis, even in the absence of external circadian cues. Interventions aimed at preserving microbial rhythms, such as time-restricted feeding (TRF), which provides food only during a defined window of the day, or stabilization of light–dark cycles, may help mitigate these effects.

#### Diet influences gut microbial rhythms

Dietary composition is another strong determinant. High-fat diets (HFDs), even when consumed during the normal feeding window, lead to a loss of microbial rhythmicity. In mice consuming an HFD, daily fluctuations in the abundance of *Firmicutes* and *Bacteroidetes* are dampened or lost [110]. In contrast, standard chow diets support robust oscillations in both taxonomic composition and microbial gene content. TRF (8-h daily window in dark/active phase) under an HFD restores microbial transcript rhythmicity, but the recovered cycles differ from those seen under normal chow, suggesting that TRF induces a distinct and feeding-dependent rhythmic profile rather than fully reinstating the original microbial oscillations [111]. Consistent with these findings, mice fed an HFD exhibit reduced microbial oscillations and decreased production of SCFAs, accompanied by an increase in hydrogen sulfide. This occurs even without overt circadian disruption, highlighting the direct impact of diet on microbial metabolism [112]. Similarly, HFD consumption alters host clock gene expression, specifically reducing *Bmal1* and *Per2* in the liver and fat, and promots feeding during the rest phase, further blunting host and microbial rhythmicity [113]. In models involving circadian disruption, such as a 12-h shift in the light–dark cycle, HFD combined with circadian misalignment leads to reduced micro­bial diversity and altered composition, with changes in *Firmicutes, Ruminococcaceae*, and *Lactobacillus* [114].

On the other hand, diets enriched in fiber may help reinforce rhythmicity. Studies show that SCFA administration could phase advance peripheral clocks and that high-fiber diets accelerate feeding-induced entrainment of the circadian system [5, 115]. These findings suggest that in addition to feeding time and composition, microbial metabolic outputs, such as SCFAs, can feed back into the host circadian system, forming a bidirectional ­regulatory loop.

#### Feeding time regulates gut microbial rhythms

Among all environmental factors, feeding time exerts the most direct influence on microbial rhythmicity. In wild-type mice, unrestricted access to food allows feeding to naturally occur during the dark phase. In this context, both microbial abundance and microbial gene expression peak during the active feeding phase. However, when feeding is restricted to the light (rest) phase, microbial oscillations shift accordingly, demonstrating that gut microbes respond acutely to nutrient availability [112]. Changing feeding time can be broadly categorized into two approaches: intermittent feeding (cessation of feeding for a period followed by *ad libitum* access) and TRF [116]. TRF has been shown to rescue the loss of microbial rhythmi­city caused by an HFD and to alleviate the associated obesity phenotype in mice [110]. In humans, TRF (providing food from 19:30 to 03:30) increases the abundance of beneficial taxa, such as *Prevotellaceae* and *Bacteroidaceae*, and enhances circadian clock gene expression through sirtuin 1 (SIRT1) activation, ultimately improving dyslipidemia [117]. TRF (8-h daily window in the dark/active phase) restores microbial rhythms by creating a high-contrast daily nutrient cycle, characterized by rhythmic availability of dietary substrates during the feeding phase and their absence during fasting [110]. Notably, TRF (8-h daily window in the dark/active phase) can partially restore the diurnal oscillations of certain gut microbes in *Per1/2*^−/−^ mice. Many gut bacteria respond directly to the host feeding–fasting cycle including *Alistipes*, *L. reuteri*, *Candidatus Arthromitus*, and Ruminococcaceae family [69]. Furthermore, TRF has been linked to increased abundance of *A. muciniphila*, suggesting a pathway for circadian alignment and metabolic benefits [118]. Feeding time and core clock genes are both critical regulators of intestinal circadian rhythms, but their mechanisms of action are not entirely overlapping. Disruption of core clock genes often leads to a complete loss of rhythmicity, highlighting their essential role in generating endogenous rhythms. In contrast, feeding time is more effective in shaping the phase and amplitude of these rhythms, acting as a potent external cue that entrains intestinal and microbial oscillations.

#### Temperature shapes gut microbial rhythms

In addition to the diet, ambient temperature and core body temperature exert significant influence on the composition and rhythmicity of the gut microbiota. In *Mongolian gerbils*, repeated intermittent exposure to low (5 °C) or high (37 °C) temperatures alternating with room temperature (23 °C) induces dynamic changes in both α- and β-diversity of gut microbial communities. Specific genera such as *Butyricimonas*, *Ruminococcus*, and *Lactobacillus* exhibit temperature-dependent fluctuations. These microbial shifts are closely correlated with host metabolic phenotypes, including resting metabolic rate, core body tempera­ture, and circulating thyroid hormone levels. Antibiotic-induced depletion of gut microbiota impairs the host thermogenic capacity and survival under cold exposure, while supplementation with propionate, a microbial metabolite, partially rescues thermoregu­lation, highlighting the role of microbial metabolites in thermal adaptation of the host [119]. Also, in growing rabbits, seasonal ambient temperature shifts not only alter cecal bacterial composition but also influence the circadian rhythm of gut microbiota. Notably, daytime feeding of rabbits under hot summer conditions increases the abundance of conditionally pathogenic genera such as *Desulfovibrio* and *Alistipes*, while reducing both the number and amplitude of rhythmic bacterial amplicon sequence variants (ASVs). In contrast, nighttime feeding enhances microbial rhythmicity and increases the predicted functional activity of bacterial metabolic pathways involved in amino acid and secondary metabolite biosynthesis. These findings suggest that elevated ambient temperature combined with mistimed feeding can disrupt microbial rhythmicity and impair gut health, increasing the risk of diarrhea [120].

Together, these studies emphasize the strong influence of environmental factors on gut microbial rhythmicity, especially light exposure, feeding time, diet composition, and ambient temperature. Disruptions such as jet lag, irregular light–dark cycles, and HFDs impair the composition of the microbiota, effects that can be transmitted to germ-free hosts and contribute to metabo­lic dysfunction. Among these factors, feeding time is the most potent regulator, capable of restoring microbial rhythms even in clock-deficient mice. Dietary quality also plays a critical role: HFDs suppress microbial oscillations, whereas fiber-rich diets help restore them. Additionally, fluctuations in ambient and body temperature modulate microbial diversity and function, linking environmental cues to microbial and host metabolic rhythms. Overall, these findings highlight the sensitivity of the microbiota to external cues and its pivotal role in maintaining host circadian and metabolic homeostasis.

### Influence of gut microbes on host rhythms

The relationship between gut microbiota and circadian rhythms is fundamentally bidirectional. On one hand, circadian clocks shape the composition, function, and daily oscillation of the gut microbiota. On the other hand, dysbiosis can disrupt host circadian gene expression, thereby impairing rhythmic physiological functions such as metabolism and immune defense.

#### Gut microbiota regulates host clock gene expression

The gut microbiota is essential for maintaining the circadian gene expression of the host. In germ-free mice, the absence of microbiota leads to disrupted rhythmic transcription of core clock genes such as *Bmal1*, *Per1*, *Per2*, and *Rev-erbα* in both the intestine and other peripheral organs such as the liver [39, 121]. These findings suggest that microbial signals, likely in the form of metabolites and structural molecules, are necessary for the synchronization and maintenance of host circadian oscillations. Among these metabolites, SCFAs such as butyrate play a promi­nent role. Butyrate functions as a HDAC inhibitor and has been shown to modulate circadian transcription by altering chromatin accessibility and regulating gene expression in a time-dependent manner. Also, it can influence protein acetylation patterns in intestinal and hepatic tissues, affecting clock protein stability and transcriptional activity [122]. In addition, microbe-modified bile acids, including deoxycholic acid and chenodeoxycholic acid, can directly modulate clock gene expression in the ileum, colon, and liver. Oral administration of these bile acids has been shown to decrease *Bmal1* expression and increase *Per2* and *Per3* expression, indicating a pathway through which the microbiota modulates the host circadian network [91]. Moreover, at the signaling level, Toll-like receptors (TLRs) on IECs detect microbial molecules and integrate these signals into the rhythmic activity of pathways such as c-Jun N-terminal kinase (JNK) and inhibitor of nuclear factor kappa-B (IKKβ). This process inhibits peroxisome proliferator-activated receptor-α (PPARα)-mediated activation of *Rev-erbα*, thereby modulating the negative feedback loop of the host circadian clock [123]. In addition, the microbiota can repress *Rev-erbα* via a DC–ILC3–signal transducer and activator of transcription 3 (STAT3) signaling axis, wherein DC sensing of microbial cues promotes IL-22 production by ILC3s, leading to STAT3 activation in epithelial cells and suppression of *Rev-erbα* expression [39].

#### Gut microbiota regulates host metabolic and absorptive rhythms

The gut microbiota plays a pivotal role in aligning host metabolic and absorptive processes with circadian rhythms by modulating metabolite profiles, immune signaling, and epigenetic states in the intestine. Diurnal fluctuations in bile acids are shaped by bacterial BSH activity, which alters bile acid composition and modu­lates intestinal and hepatic FXR signaling, thereby influencing lipid and cholesterol metabolism in a time-of-day–dependent manner [124]. Targeted inhibition of the gut microbial TMA pathway protects mice from metabolic disturbances associated with diet-induced obesity or leptin deficiency by reorganizing host circadian regulation of phosphatidylcholine and energy metabolism [103].

Microbial sensing by DCs activates ILC3s to secrete IL-22, which in turn stimulates STAT3 signaling in IECs. This pathway represses the expression of *Rev-erbα*, relieving transcriptional inhibition of nuclear factor interleukin-3-regulated protein or E4 promoter-binding protein 4 (*Nfil3/E4bp4*) and thereby inducing time-of-day–specific expression of lipid absorption genes such as *Cd36* and *Fabp4*, aligning lipid uptake with feeding rhythms [39]. In parallel, microbial cues drive the rhythmic recruitment of HDAC3 to promoters of nutrient transporter and lipid meta­bolism genes, including *Cd36* and *Fabp2*, in IECs. This epigenetic regulation ensures that glucose and lipid absorption peaks during the active feeding phase, thereby synchronizing nutrient uptake with circadian metabolic demands [38]. Furthermore, *A. muciniphila* modulates host circadian glucose metabolism by restoring impaired glucose tolerance rhythms at the light–dark transition (ZT12) through improving gut microbiota composition and enhancing GLP-1 secretion in intestinal free fatty acid receptor 4 (*Ffar4*)-deficient mice [125].

Together, these findings highlight that the gut microbiota shapes host metabolic and absorptive rhythms through coordinated modulation of metabolite oscillations, immune–clock interactions, and epigenetic regulation, ensuring that nutrient uptake and energy utilization are optimally matched to daily activity cycle in the host.

#### Gut microbiota regulates host immune rhythms

The gut microbiota orchestrates diurnal mucosal immunity by aligning antimicrobial, antigen-presenting, and regulatory pathways with feeding-driven circadian rhythms. A specific group of mucosa-associated symbionts, particularly segmented filamentous bacteria (SFB), are rhythmically attached to epithelial cells at the beginning of the active phase. This rhythmic adhesion triggers innate immune responses, notably the secretion of antimicro­bial peptides (AMPs), such as REG3G, lipocalin 2 (LCN2), and S100 calcium-binding protein A8 (S100A8). Among them, REG3G peaks at ZT12, providing enhanced protection against pathogens during feeding. In mice lacking SFB, this AMP rhythm is absent until SFB are introduced through cohousing, underscoring the essential role of mucosal symbionts in shaping diurnal innate immunity [6]. Beyond their role in innate defenses, these mucosa-attached microbes also promote rhythmic MHC class II-mediated cytokine production, thereby bridging innate and adaptive immunity [68]. In the small intestine, commensals such as SFB drive diurnal oscillations in epithelial MHC-II expression, which facilitates time-of-day–specific antigen presentation to CD4^+^ T cells. This interaction coordinates with intraepithelial IL-10^+^ lymphocytes, whose cytokine output also fluctuates over the circadian cycle to reinforce barrier integrity. Disruption of this diet–microbiota–MHC class II–IL-10 axis through circadian misalignment, altered feeding schedules, or epithelial MHC-II deficiency disrupts the temporal coordination of immune responses, allowing excessive microbial antigen influx and predisposing to Crohn-like enteritis.

In contrast, during the resting/fasting phase, secretory immunoglobulin A (sIgA), a key mucosal antibody, reaches its peak. sIgA is produced by plasma cells in the lamina propria and secreted into the gut lumen, where it selectively binds and neutralizes bacteria and their toxins. This delayed response clears microbes tolerated earlier during feeding, thereby minimizing inflammation and promoting homeostasis. Interestingly, sIgA secretion follows feeding-driven, rather than clock gene-driven, rhythms. Disruption of clock gene transcription does not completely abo­lish the rhythmic pattern of sIgA, whereas reversing the feeding schedule shifts the sIgA rhythm [62].

Recent evidence also reveals that microbial and metabolic signals orchestrate mucosal immunity in a rhythmic manner. The microbiota processes rhythmic feeding signals, and promotes the TGF-β signaling via HDAC3 in epithelial cells, which in turn induces the transcription of *Pou2f3* and drives the diurnal differentiation of tuft cells [11]. These specialized chemosensory cells function as immune sentinels and contribute to just-in-time mucosal barrier defense, such as rhythmic activities of type-2 immune circuit. Another study reported that bacterial butyrate is a key molecule that mediates microbial regulation of HDAC3-dependent tuft cell differentiation [126]. Given that gut microbes can produce butyrate rhythmically [5], this suggests that butyrate may not only restrict tuft cell differentiation but also contribute to its diurnal oscillation, thereby providing the host with a dynamic, time-specific mechanism for immune regulation.

Together, these findings demonstrate that the gut microbiota entrains mucosal immune functions to diurnal rhythms.

## Circadian crosstalk between the gut and distant organs

**Figure 5:**
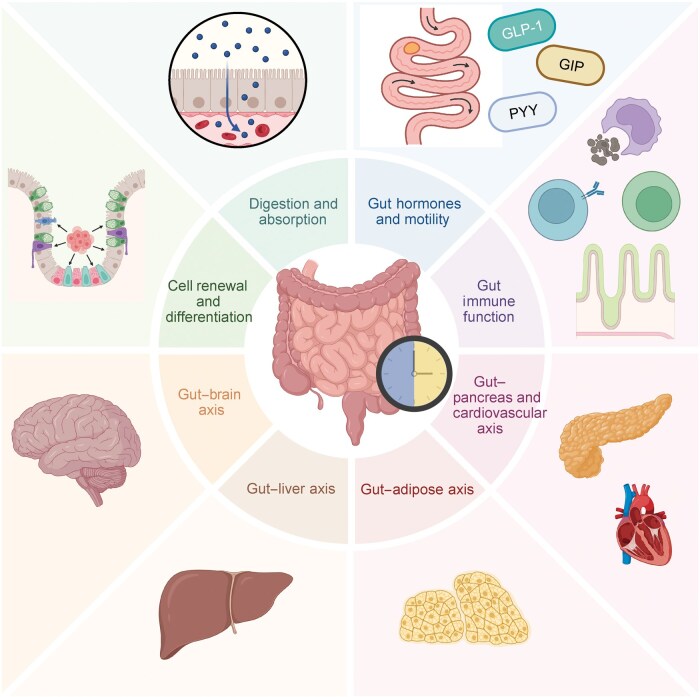
Circadian regulation of gut physiology and inter-organ communication. This diagram illustrates the ddiversephysiological processes in the gastrointestinal system that are under circadian control. Core functions such as cell renewal and differentiation, digestion and absorption, hormone secretion (e.g. GLP-1, PYY, and GIP), motility, and immune function follow daily rhythms. These intestinal rhythms not only coordinate local functions but also influence distant organs through the gut–brain, gut–liver, gut–adipose, gut–pancreas, and gut–cardiovascular axes. Through these pathways, the gut acts as a central hub linking circadian timing to whole-body metabolic and immune homeostasis.

Beyond its local functions in digestion, immunity, and barrier maintenance, the gut plays a central role in regulating the physiology of distant organs through systemic signaling, such as the brain, liver, adipose tissue, pancreas, and cardiovascular system. These inter-organ communications, collectively referred as the “gut–organ axes”, are mediated by circadian rhythms, which coordinate the timing of molecular, metabolic, neural, and immune activities across tissues. The gut communicates with these organs via hormones, microbial meta­bolites, immune signaling, and neural circuits, each of which displays 24-h oscillations. These rhythms are entrained by feeding time and environmental light, allowing the gut to act as both a sensor and synchronizer of systemic physiology. This chapter explores how the gut interacts rhythmically with peripheral organs through the gut–brain, gut–liver, gut–adipose, gut–pancreas, and gut–cardiovascular axes, highlighting how circadian misalignment can lead to metabolic and inflammatory disorders, and pointing to novel therapeutic strategies based on chrono-nutrition and circadian medicine (Fig. 5).

### Circadian regulation in the gut–brain axis

Microbial neurotransmitters such as GABA and serotonin influence brain rhythmicity; here we further explore their role in gut–brain communication. The gut–brain axis is a bidirectional communication system linking the gastrointestinal tract with the central ner­vous system, mediated through neural, endocrine, immune, and microbial pathways. Circadian rhythms are deeply embedded in this network, aligning gut and brain functions with environmental cycles such as light and feeding. Notably, both the gut and the brain possess intrinsic clocks, and their synchronization is essential for maintaining systemic homeostasis.

In Alzheimer’s disease (AD), circadian disruption and gut dysbio­sis co-occur, with evidence for increased intestinal permeability and neuroinflammation [127]. Conversely, microbiota ­depletion alters circadian transcriptional programs in the SCN and stress axis; microbial rhythmicity is required for appropriate brain circadian responses [128]. This feedback loop is further supported by the evidence that SCFAs modulate central immune tone and neuronal activity via microglial and macrophage signaling, while tryptophan derivatives regulate neurodevelopment and circadian rhythmic expression of immune genes via AhR pathways [129]. In mouse models, recolonization with specific bacteria such as *Limosilactobacillus reuteri* restores corticosterone rhythmicity and gene expression in brain stress circuits, directly demonstrating that the presence of the microbiota, and potentially their rhythmic abundance, is essential for circadian regulation of stress responses [128].

### Circadian regulation in the gut–liver axis

The gut–liver axis connects the intestine and the liver through the portal circulation, bile acid signaling, and immune-metabolic crosstalk. This axis plays a pivotal role in coordinating digestion, detoxification, and metabolic regulation. Both the gut and the liver harbor robust circadian clocks, and their temporal synchronization is crucial for maintaining metabolic homeostasis.

Bile acid synthesis and hepatic metabolism follow robust circadian patterns tightly controlled by gut–liver feedback. Diurnal variations in bile acid levels and synthetic enzyme activity ­ensure that nutrient processing aligns with feeding cycles [130]. In mice, the expression of *Cyp7a1*, the key enzyme for bile acid synthesis, rises during light phase and peaks near the onset of the dark (­active/feeding) phase; circulating bile acids also show pronounced ­diurnal peaks around the active phase [131]. These bile acids not only facilitate lipid absorption but also act as signaling molecules, activating receptors such as FXR and TGR5 in both the liver and the intestine to regulate glucose metabolism, inflammation, and insulin sensitivity [132]. The gut microbiota influence bile acid composition by converting primary bile acids into secondary bile acids, thereby modulating the activation of hepatic receptors. Antibiotic-induced dysbiosis reduces hepatic/ileal FXR expression, and perturbs bile acid signaling and metabolism in rodents [133].

Circadian misalignment due to shift work, irregular feeding, or sleep disruption uncouples gut–liver rhythms. In mouse models, compared with HFD-*ad libitum*, HFD-TRF (8-h daily window in the dark/active phase) restores the rhythmic expression of hepatic clock genes, improves bile acid cycling, and ameliorates features of nonalcoholic fatty liver disease (NAFLD) and metabolic syndrome [134].

Overall, the gut–liver axis exemplifies how peripheral circadian clocks coordinate organ function through shared metabolic and hormonal signals. The liver clock regulates bile acid production, inflammatory tone, and nutrient metabolism, while gut microbial metabolites provide feedback to fine-tune hepatic rhythms. Disruption of this bidirectional system contributes to metabolic disorders such as NAFLD, T2D, and systemic inflammation.

### Circadian regulation of the gut–adipose axis

The gut and adipose tissue engage in dynamic bidirectional communication, forming a gut–adipose axis that is critical for maintaining systemic metabolic balance. Recent evidence suggests that this axis is under circadian control, with gut-derived hormonal and microbial signals modulating adipose tissue rhythms, while adipose clocks in turn influence systemic energy homeostasis. Disruption of this rhythmic crosstalk contributes to obesity, insulin resistance, and chronic inflammation.

Gut-derived hormones such as GLP-1 and PYY exhibit diurnal secretion patterns that regulate lipid storage and energy expendi­ture in adipose tissue. GLP-1, secreted from intestinal L cells in response to feeding, not only enhances insulin secretion but also reduces appetite and promotes lipid oxidation. Its plasma levels peak during the active phase in humans and mice, aligning with feeding windows and postprandial metabolic demands [43, 47]. PYY follows a similar rhythm, reinforcing satiety signals and limiting fat accumulation during the feeding phase.

SCFAs act as time-stamped metabolic cues linking gut fermentation rhythms with adipose energy balance (also see Section “SCFAs”). SCFAs activate G-protein coupled receptors (GPR41 and GPR43) in adipocytes and immune cells to modulate lipolysis, thermogenesis, and anti-inflammatory pathways [135, 136]. High SCFA levels promote energy expenditure and suppress adipose inflammation, functioning as key time-stamped signals for metabolic adaptation. Conversely, TRF restores rhythmic coordination across the gut–adipose axis, improves SCFA production, ­enhances *Bmal1* expression in adipose tissue, and protects against metabolic diseases [137].

In summary, the gut–adipose axis integrates circadian signals from dietary intake, microbial metabolism, and host clock machinery. Rhythmic secretion of gut hormones and ­microbial metabolites entrains adipose tissue clocks to promote lipid homeostasis and suppress inflammation. Disruption of this coordination impairs energy balance and promotes cardiometabolic disease, highlighting the therapeutic potential of restoring gut–adipose circadian alignment through nutritional and beha­vioral interventions.

### Circadian regulation in the gut–peripheral endocrine and cardiovascular axes

Beyond its local functions, the intestine plays a critical role in regu­lating peripheral organ physiology via circadian coordination of endocrine and metabolic signals. Among the most well-studied systems influenced by gut rhythmicity are the cardiovascular and pancreatic axes. These systems receive rhythmic inputs from gut-derived metabolites and hormones, which help synchronize peripheral clocks with feeding cycles and environmental cues.

#### Gut–cardiovascular axis: microbial rhythms influence heart and vascular function

The cardiovascular system exhibits intrinsic circadian rhythms in blood pressure, heart rate, and vascular tone, driven by both central and peripheral clocks. Recent findings suggest that the gut microbiota contributes to cardiovascular rhythmicity through the production of metabolites such as SCFAs, immune modulation, and vagal nerve signaling.

In mouse models of myocardial infarction (MI), microbiota depletion impairs cardiac repair, increases inflammation, and exa­cerbates adverse ventricular remodeling. Restoration of gut flora or implementation of TRF improves outcomes—but only in mice with intact circadian clocks. TRF aligned with the active phase improves post-MI cardiac outcomes via circadian-mediated gut responses [138].

Human studies support a similar link. The circadian profiles of blood pressure correlate with gut microbiota composition: bene­ficial genera such as *Lactobacillus* and *Alistipes* are enriched in individuals with normal diurnal blood pressure fluctuations, while dysbiotic taxa like *Prevotella* are prevalent in patients with blunted rhythms. Circulating acetate levels negatively correlate with 24-h blood pressure variability, suggesting that microbial metabolite rhythmicity plays a role in cardiovascular timing [139, 140]. Mouse studies also show that SCFAs can regulate blood pressure and reduce hypertension via olfactory receptor 78 and GPR41 [141, 142].

#### Gut–pancreas axis: hormonal timing regulates glucose homeostasis

The pancreas, as a central regulator of metabolic homeostasis, also exhibits circadian control over both exocrine (digestive enzyme) and endocrine (hormone) functions. Key hormones such as insulin, glucagon, and amylin show daily rhythmic secretion, which is tightly coordinated with feeding behavior and intestinal signals.

Furthermore, pancreatic insulin and glucagon levels exhibit daily rhythms in antiphase: insulin peaks during the postprandial period, while glucagon rises during fasting. These hormonal dynamics are regulated not only by local pancreatic clocks but also by feedback to central pathways such as the hypothalamus, forming a closed gut–pancreas–brain circuit. Disruptions in feeding timing or clock gene function impair these rhythms, contributing to insulin resistance, weight gain, and an increased risk of T2D [143].

Together, circadian rhythms orchestrate a network of bidirectional signals between the gut and distant organs, ensuring temporal coordination of metabolic, immune, and neuroendocrine functions. All axes described above rely on rhythmic cues such as feeding cycles, microbial metabolites, and peripheral clocks to maintain organ-specific and systemic homeostasis. Disruption of this rhythmic crosstalk, whether due to shift work, irregular eating, or genetic clock mutations, leads to desynchronization of metabolic pathways, impaired immune responses, and increased disease susceptibility. Understanding the temporal structure of these inter-organ communications opens the door to chrono-therapeutic strategies such as TRF, microbiota modulation, and clock-targeted medicines to restore circadian alignment and improve systemic health.

## Gut rhythms and human disease

### Gut rhythm disruptions in inflammatory and neoplastic gut diseases

**Figure 6:**
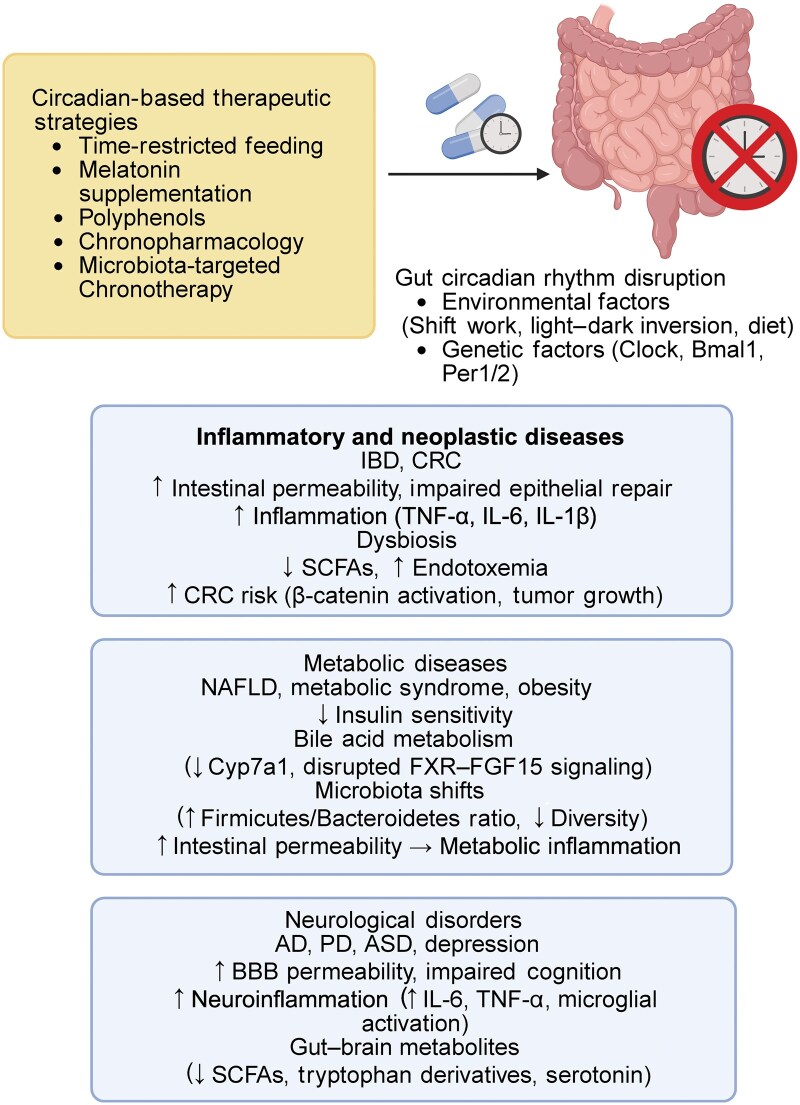
Gut circadian rhythm disruption and disease associations. Environmental (shift work, light–dark inversion, and diet) and genetic (*Clock*, *Bmal1*, and *Per1/2*) disruptions impair gut circadian rhythms, leading to increased intestinal permeability, inflammation, and dysbiosis. These changes contribute to inflammatory and neoplastic diseases (IBD and CRC) and metabolic disorders (NAFLD, obesity, and metabolic syndrome) via altered bile acid metabolism and microbiota shifts, and neurological disorders (AD, PD, ASD, and depression) through BBB disruption, neuroinflammation, and reduced gut–brain metabolites. Circadian-based therapeutic strategies such as TRF, melatonin, polyphenols, chronopharmacology, and microbiota-targeted chronotherapy may help restore rhythmicity and improve disease outcomes.

Circadian rhythms are essential regulators of gastrointestinal homeostasis, orchestrating epithelial renewal, immune surveillance, and microbial interactions. Disruptions of these rhythms—due to environmental or genetic factors—are increasingly recognized as contributors to various gastrointestinal diseases, including inflammatory bowel disease (IBD), irritable bowel syndrome (IBS), and colorectal cancer (CRC) [144–147] (Fig. 6).

In IBD, circadian misalignment aggravates intestinal inflammation by impairing the gut epithelial barrier and skewing immune responses. Studies using *Clock* and *Bmal1* knockout mouse mo­dels have demonstrated that the absence of core circadian genes leads to increased intestinal permeability, delayed epithelial regeneration, and enhanced susceptibility to dextran sulfate so­dium (DSS)-induced colitis [148, 149]. These mice exhibit elevated levels of pro-inflammatory cytokines such as TNF-α, IL-6, and IL-1β, along with disrupted expression of tight junction proteins, revealing a central role of the circadian clock in maintaining mucosal integrity [150].

Moreover, circadian rhythm disruption affects the composition and temporal dynamics of gut microbiota, promoting dysbiosis that further impairs immune balance. In mouse models subjected to chronic light–dark phase shifts, microbial community composition loses its daily oscillation, and colitis severity worsens in parallel with reduced SCFA levels and increased endotoxemia [114, 151]. This microbiota–immune–clock feedback loop has also been observed in human IBD patients, who frequently suffer from sleep disturbances and demonstrate altered clock gene expression in intestinal tissues [152].

Beyond inflammation, circadian misalignment may increase the risk of CRC. Chronic inflammation, impaired epithelial repair, and DNA damage accumulation underlie this link. In mice with *Per2* deficiency, enhanced tumor growth and increased β-catenin signaling have been observed in azoxymethane (AOM)/DSS-induced CRC models [153]. Thus, disruption of gut circadian control not only triggers inflammatory diseases but also creates a tumor-promoting microenvironment.

Clinical evidence supports these findings: patients with shift work histories or sleep dysfunctions have higher IBD and CRC incidence rates, and time-based interventions, such as light therapy or melatonin, may help restore mucosal rhythmicity and alleviate disease severity [154].

In an IBD model, Amara *et al*. exposed mice to DSS-induced colitis along with circadian rhythm inversion every 5 days for 3 months. They observed that circadian disruption significantly exacerbates DSS-induced microbial dysbiosis and colitis severity. Specifically, microbial diversity is markedly reduced, with lower Shannon and Chao1 indices, and the Firmicutes/Bacteroidetes ratio declines from 1.78 in controls to 1.36 in the DSS + circadian disruption group. At the genus level, *Lactobacillus*, *Alloprevotella*, *Turicibacter*, and *Streptococcus* are elevated in disrupted groups, while beneficial taxa such as *Lachnospiraceae NK4A136* are dimi­nished. These shifts are accompanied by worsened intestinal inflammation and impaired barrier integrity, highlighting circadian disruption as an important environmental factor that can aggravate gut dysbiosis and IBD progression [151].

### Gut rhythm disruptions in metabolic diseases

Circadian rhythms are central regulators of metabolic homeostasis. They coordinate nutrient sensing, hormone secretion, lipid turnover, and energy expenditure across multiple organs. Disruption of circadian rhythms through genetic mutations, sleep deprivation, or irregular feeding can disturb these finely tuned processes and contribute to the development of metabolic diseases such as T2D, obesity, NAFLD, and metabolic syndrome [8, 155, 156] (Fig. 6).

One key mechanism involves circadian disruption impairing bile acid synthesis and enterohepatic circulation. *Clock* mutations interfere with hepatic *Cyp7a1* expression and intestinal FXR–fibroblast growth factor 15 (FGF15) signaling, contributing to cholesterol accumulation, lipid dysregulation, and hepatic steatosis [157]. Both genetic (*Per1/Per2* knockout) and environmental (sleep loss) models confirm that clock disruption alters bile acid metabolism and lipid synthesis, leading to liver damage and NAFLD [158]. These molecular disruptions are exacerbated by HFDs and irregular eating patterns, which further desynchronize central and peripheral clocks and promote metabolic inflammation.

Gut microbiota also plays an important role in metabolic disease. For example, transferring the microbiota from feces of jet-lagged humans to germ-free mouse recipients was sufficient to induce glucose intolerance and increase adiposity, indicating a causal role of microbiota rhythmicity in host metabolism [69]. In a dietary circadian disruption model, Voigt *et al*. exposed C57BL/6J mice to weekly reversals of the light–dark cycle (simulating jet lag or shift work) and fed them with either standard chow or a high-fat, high-sugar Western diet. They found that circadian disruption alone does not significantly alter gut microbiota under standard diet conditions. However, when combined with a Western diet, it leads to pronounced microbial dysbiosis, characterized by an increase in Firmicutes, a reduction in Bacteroidetes, and a higher Firmicutes/Bacteroidetes ratio. Microbial diversity, assessed by the Shannon index, also declines. Notably, *Ruminococcus* is enriched, while *Desulfosporosinus* and *Desulfotomaculum* are depleted. This dysbiosis is associated with increased intestinal permeability and disruption of tight junction proteins such as occludin, suggesting a potential link between circadian misalignment, gut barrier dysfunction, and chronic inflammatory or metabolic diseases such as obesity and metabolic syndrome [114].

### Gut rhythm disruptions in neurological disorders

An expanding body of evidence supports a bidirectional link between the gut and the brain, with circadian rhythms serving as a temporal framework for the gut–brain axis. Disruption of this temporal alignment can impair blood–brain barrier (BBB) integrity, trigger neuroinflammation, and alter neurotransmitter metabolism, collectively increasing susceptibility to neurological, psychiatric, and developmental disorders such as AD, Parkinson’s disease (PD), depression, and autism spectrum disorder (ASD) [159–161] (Fig. 6).

At the molecular level, circadian rhythm governs the activity of tight junction proteins in the BBB and modulates neuroimmune responses through clock-controlled genes such as *Per2*, *Rev-erbα*, and *Nfil3*. In *Rev-erbα*-deficient mice, elevated IL-6 and TNF-α expression in the hippocampus, impaired BBB permeability, and enhanced microglial activation—all hallmarks of neuroinflammation—have been observed. Similarly, circadian disruption accelerates microglial reactivity and synaptic pruning, which are key mechanisms in neurodegenerative diseases [159]. Moreover, long-term circadian disruption via chronic light exposure or reversed feeding cycles has been shown to reduce *Per2* expression in the hippocampus and cortex, impair cognition, and increase amyloid deposition in AD mouse models [162].

The gut microbiota mediates many of these effects. In AD patients, *Bacteroidetes* are notably increased, whereas *Bifidobacterium* and *Firmicutes* are decreased. In PD patients, *Acidaminococcus*, *Akkermansia*, *Anaerotruncus*, *Bilophila*, *Christensenella*, *Lactobacillus*, *Streptococcus*, and *Turicibacter* are markedly elevated [163, 164]. Gut microbiota produces neuroactive metabolites such as SCFAs, serotonin (5-HT), and tryptophan-derived indoles, which influence the central ner­vous system functions. Disruption of circadian rhythms alters the abundance of microbiota involved in these pathways. For instance, acute sleep deprivation reduces gut microbe-derived SCFA levels and increases gut permeability, resulting in elevated systemic and brain inflammation. In ASD mouse models, these changes correlate with behavioral impairments, suggesting that microbial signals under circadian control may regulate emotional and cognitive outcomes [165].

### Circadian-based therapeutic strategies

Mounting evidence supports the application of circadian-based interventions (chronotherapy) in managing chronic diseases. These strategies aim to restore or exploit biological rhythms to optimize treatment outcomes by synchronizing therapeutic actions with natural oscillations of the body in physiology, hormone secretion, immune activity, and metabolism [166] (Fig. 6).

One of the most established chronotherapeutic approaches is TRF. In rodents, limiting food access to an 8–9-h window during the active phase reinforces circadian clock oscillations and reverses HFD-induced metabolic dysfunction including obesity, T2D, hyperlipidemia, fatty liver, and inflammation [137, 167]. Consistently, restoring peripheral rhythmicity with TRF norma­lizes body weight and glucose metabolism even under constant darkness in SCN-specific *Bmal1*-deficient mice [168]. Human data parallel these findings: in prediabetic men, early TRF (6-h eating window and 18-h fast) improves insulin sensitivity, blood pressure, and oxidative stress without weight loss; in overweight se­dentary men, TRF enhances nocturnal and postprandial glycemic control as well as overall well-being [169, 170].

Melatonin supplementation represents another effective intervention, particularly for neurological and inflammatory disorders. Melatonin, a hormone with a well-established role in circadian regu­lation, has anti-inflammatory and antioxidant properties. In clinical trials, it improves sleep quality and reduces pro-inflammatory cytokines in patients with IBD, and slows cognitive decline in patients with AD and PD [171]. Melatonin supplementation can also effectively prevent and treat intestinal injury and inflammation induced by acute sleep deprivation by restoring gut microbiota balance and enhancing mucosal barrier function. It increases beneficial bacteria such as *Faecalibacterium*, promotes the production of the anti-inflammatory metabolite butyrate, upregulates the butyrate transporter, suppresses NF-κB/NACHT, LRR and PYD domains-containing protein 3 (NLRP3)-mediated inflammation, and enhances both antioxidant capacity and tight junction protein expression. This dual regulation of the “microbiota–barrier–immune” axis positions melatonin as a promising therapeutic agent for gut disorders associated with circadian disruption and sleep loss [172]. Dietary polyphenols are plant-derived compounds with potent antioxidant and anti-inflammatory activities. It modulates gut microbiota composition, enhancing beneficial bacteria and SCFA production to influence host circadian rhythms and alleviate disease. Beneficial microbes such as *Lactobacillus*, *Bifidobacterium*, and *Faecalibacterium prausnitzii* enhance intestinal barrier integrity by upregulating tight junction proteins such as occludin and claudin-1 and stimulating mucin production, while reducing inflammation through butyrate generation [173].

Chronopharmacology, the practice of aligning medication timing with biological rhythms, is being increasingly adopted in metabolic medicine. For example, giving low-dose SGLT2 inhibitors in the evening exploits nocturnally low SGLT2 expression to improve glycemic control [174], while evening antihypertensives leverage the physiologic nighttime dip in blood pressure to lower the risk of MI and stroke in patients [175]; bedtime administration of slow-release corticosteroids more effectively blunts morning flares and pain in rheumatoid arthritis [176]. Beyond host targets, timing can also shape the gut microbiota and downstream clinical events: synchronizing antibiotic administration with the feeding phase may reduce antibiotic-associated diarrhea and *C. difficile* overgrowth, while delivering microbiota-active nutrients during the active/feeding phase can amplify SCFA oscillations, thereby strengthening barrier rhythms and reducing endotoxemia-related inflammation.

Looking forward, precision chronomedicine aims to integrate individual circadian profiles, microbial rhythms, and genetic background to develop personalized, time-based treatments. As our understanding deepens, aligning therapy with the “when” of physiology, not just the “what” or “how,” may prove essential in the prevention and treatment of complex diseases.

## Limitations and future perspectives

Biological variables intrinsic to the host, such as sex and age, are increasingly recognized as modulators of gut microbial circadian rhythms [177]. However, current evidence is largely derived from young adult mouse models, leaving the extent, mechanisms, and translational relevance of these effects in humans and across the lifespan insufficiently defined. In mice, total bacterial load and several major microbial groups including *Bacteroidetes*, *Firmicutes*, *Lactobacillaceae*, and *Clostridiales* exhibit stronger diurnal oscillations in females compared with males [70]. These rhythms disappear in both sexes when the circadian gene *Bmal1* is deleted at the whole-body level, suggesting that sex-dependent microbial oscillations arise from shared upstream circadian mechanisms in the host. Beyond changes in community composition, sex-specific daily patterns are also observed in microbial metabolic pathways, including polysaccharide degradation and amino acid metabolism, as well as in microbial metabolites such as SCFAs. These microbial rhythms influence the temporal expression of metabolic and immune-related genes in the host and generate sex-specific daily variation in metabolic and immune functions [178]. In addition, sex-dependent differences have been observed under metabolic stress. For example, females show more pronounced rhythmic alterations in clock gene expression and microbial composition in the ileum, whereas males display stronger rhythmic changes in the liver during activity-based ­anorexia [179]. Together, these findings establish sex as an important biological variable in shaping gut and microbial circadian rhythms. However, nearly all existing data arise from mouse mo­dels, and whether comparable sex-related rhythmic differences exist in humans remains to be determined.

Age represents another biological characteristic that may influence circadian regulation in the gut, yet current evidence remains limited. Most studies have focused exclusively on young adult animals, and systematic comparisons among early-life, young adult, middle-aged, and aged groups are lacking. High-resolution temporal sampling has not been conducted across different age groups, leaving it unclear whether older individuals show reduced rhythmic amplitude, weakened microbial oscillations, or altered daily patterns of microbial metabolites. Understanding age-related effects on gut microbial rhythms will likely require long-term monitoring of model organisms under controlled dietary and environmental conditions, so that external rhythmic cues do not dominate the signal of interest. In humans, this question is even more challenging to address because diet, lifestyle, and environmental exposures vary substantially among individuals. As a result, the potential contribution of age to circadian regulation along the gut–microbiota axis remains an open and important research gap. Future work should incorporate longitudinal and demographically diverse human cohorts to define the heterogeneity and population specificity of microbial and host circadian rhythms. Such advances would support the development of more precise and personalized circadian interventions.

Antibiotic treatment poses a substantial risk to microbial rhythmicity and downstream host physiology. Chronic broad-spectrum antibiotic exposure drastically depletes microbial biomass, disrupts metabolite profiles, and eliminates serotonin and vitamin B_6_ synthesis in the gut, both of which are closely tied to circadian re­gulation of brain and intestinal function [180]. These disruptions in neurotransmitter-related pathways are accompanied by altered sleep architecture in mice, indicating a systemic impact through the microbiota–gut–brain axis. The loss of rhythmic microbial metabolites may thereby contribute to circadian misalignment at the organismal level, further emphasizing the need to limit unnecessary antibiotic use, especially in the context of rhythm-sensitive conditions.

Coffee, a common dietary factor consumed by humans, may influence gut microbial rhythms through its bioactive components. While coffee itself does not ameliorate microbial dysbiosis in metabolic syndrome mice, caffeine, and chlorogenic acid partially improve the disrupted SCFA profile by increasing propionate and butyrate levels, both of which are rhythmically produced and play roles in host metabolic timing [181]. Although coffee ­induces only limited changes at the genus level in the gut microbiota, its bioactive components may modulate the circadian rhythms of gut-derived metabolites via microbial metabolism. This suggests a modest but potential role for coffee in supporting gut rhythmicity—possibly through host–microbe metabolic crosstalk rather than direct microbial entrainment.

An emerging concern is the influence of microplastics (MPs) and nanoplastics (NPs) on gut rhythms, particularly through their disruption of *Akkermansia* and SCFA-related pathways. Studies show that polystyrene NPs impair gut barrier integrity by disrupting goblet cell function and suppressing the expression of mucin (MUC13) and tight junction proteins such as ZO-1. This damage increases intestinal permeability and reshapes microbial composition, including the enrichment of pro-inflammatory taxa like *Ruminococcaceae* [182]. Given that mucosal *Akkermansia* and SCFA production exhibit circadian patterns, disruption of the mucus niche and microbial signaling likely leads to desynchronization of host–microbe rhythmicity. Furthermore, MPs are ubiquitous and can be ingested via food, water, and even household utensils, and have been associated with oxidative stress, endocrine disruption, and microbial gene dysregulation, raising broader concerns about their chronic effects on circadian homeostasis [183]. Taken together, from antibiotics and human-specific dietary factor to MPs and NPs, these exogenous exposures underscore how readily host–microbe rhythmicity can be perturbed. Accordingly, future studies should employ human-relevant models and incorporate time-of-day protocols to accurately capture circadian behavior.

However, current evidence, based largely on nocturnal rodents under artificial laboratory light schedules, may not fully translate to diurnal humans. One major limitation arises from the predo­minant use of rodent models, particularly nocturnal species such as mice, whose behavioral and molecular circadian rhythms differ substantially from those of diurnal humans. For example, key clock proteins such as PER and NR1D1 have opposite phase between mice and human prefrontal cortex [184], and in the gut, this shifting of the rhythmic phase may also cause difference in interpreting mechanisms. While murine models have been indispensable for dissecting core clock mechanisms, future research must integrate human-relevant platforms such as intestinal organoids and *ex vivo* tissue culture systems. Recent advances in human intestinal organoid technology have begun to reveal clock-driven transcriptional rhythms in human epithelial cells, offering pro­mising avenues to model human gut rhythmicity more accurately. Even when moving beyond rodents, translation is further constrained by pronounced interindividual variation in human circadian timing.

Another challenge is the substantial interindividual variability in circadian rhythms driven by differences in lifestyle and environmental exposure, including dietary patterns, activity schedules, and sleep–wake behavior. These variables interact with modern lifestyle factors to create heterogeneous circadian profiles across the population, such as shift work, jet lag, and late-night eating. Consequently, establishing generalizable models of circadian regu­lation is difficult. Large-scale cohort studies and wearable circadian monitoring technologies may help stratify individuals based on rhythmic phenotypes and tailor interventions accordingly. These human-level behavior differences interact with artificial laboratory environments, making fixed LD 12:12 protocols a poor proxy for real-world photoperiods.

Most circadian rhythm experiments rely on a fixed LD 12:12 protocol, where organisms are exposed to exactly 12 h of light followed by 12 h of darkness. While this design standardizes laboratory conditions, it does not reflect the real-world variability in day length. Beyond this, natural transitions between light and dark are gradual, occurring as dawn and dusk, whereas experimental paradigms tend to use abrupt light–dark switches that lack ecological validity. Moreover, the brain master clock, the SCN, is sensitive to the length of photoperiod [185]. Studies have shown that variations in day length modulate the SCN synchronization properties, such as phase angle and amplitude of entrainment. Together, these findings highlight significant limitations in the standard LD 12:12 model when aiming to understand circadian behavior in natural settings. To better translate circadian research into clinical and real-world applications, it is essential to incorporate seasonally variable day lengths and simulate gradual light transitions. Without ecologically valid light−feeding contexts, it becomes difficult to resolve the true temporal architecture of clock−signaling crosstalk.

At the molecular level, the mechanistic crosstalk between core clock genes and key signaling pathways, including Wnt, JNK, phosphatidylinositol 3-kinase/protein kinase B (PI3K/AKT), mammalian target of rapamycin (mTOR), and TLR, is not fully understood. While studies have shown that BMAL1 can transcriptionally regulate Wnt components in stem cells [186], and that clock genes impact mTOR activity in immune cells [187], few have systematically mapped the bidirectional feedback loops or explored temporal dynamics. Systems biology approaches, coupled with CRISPR-based perturbation screens, may help deli­neate these regulatory architectures in a cell-type-specific manner. Clarifying these clock signaling pathway feedbacks at the cellular level should proceed in parallel with causal *in vivo* tests of microbe-derived temporal signals.

The gut microbiota is increasingly recognized as both an output and modulator of circadian rhythms, yet many studies remain correlative. To clarify causality, recent work has utilized antibio­tic depletion, germ-free models, and time-restricted microbial reconstitution to define how microbial metabolites entrain host clocks. Integrating multi-omics data, particularly metagenomics, metabolomics, and single-cell transcriptomics across time points, could reveal microbial rhythmicity with high functional resolution. Only through mechanistic and causal resolution can we define disease-specific chronotherapeutic windows and tailor interventions for individual chronotypes.

Finally, although circadian-based interventions such as TRF, melatonin therapy, or light modulation have shown promise in preclinical and pilot human studies, a key barrier to clinical translation is the lack of defined chronotherapeutic windows, that is, the optimal time periods during which interventions are most effective. It remains unclear whether different diseases require distinct timing strategies, and whether circadian therapies must be aligned with individual chronotypes (e.g. early versus late sleepers). Personalized chronotherapy, which considers individual circadian profiles and molecular clocks, represents a future direction but requires more mechanistic validation. Advances in wearable circadian diagnostics, along with time-series modeling, may eventually support the implementation of precision circadian medicine for gastrointestinal and systemic diseases.
